# Genome-wide CRISPR screen identifies Menin and SUZ12 as regulators of human developmental timing

**DOI:** 10.1038/s41556-025-01751-5

**Published:** 2025-09-02

**Authors:** Nan Xu, Hyein S. Cho, James O. S. Hackland, Silvia Benito-Kwiecinski, Nathalie Saurat, Oliver Harschnitz, Marco Vincenzo Russo, Ralph Garippa, Gabriele Ciceri, Lorenz Studer

**Affiliations:** 1 The Center for Stem Cell Biology and Developmental Biology program; Memorial Sloan Kettering Cancer Center, New York, NY USA; 2 Louis V. Gerstner Jr. Graduate School of Biomedical Sciences, New York, NY USA; 3 Gene Editing and Screening Core Facility, Memorial Sloan Kettering Cancer Center, New York, NY USA; 4 Present Address: Australian Regenerative Medicine Institute, Monash University, Clayton, Australia; 5 Present Address: Human Technopole, Milan, Italy; 6 Present Address: Sylvester Comprehensive Cancer Center, Miller School of Medicine, University of Miami, Miami, FL USA

**Keywords:** CRISPR-Cas systems, Stem-cell differentiation, Embryonic stem cells, Differentiation, High-throughput screening

## Abstract

Embryonic development follows a conserved sequence of events across species, yet the pace of development is highly variable and particularly slow in humans. Species-specific developmental timing is largely recapitulated in stem cell models, suggesting a cell-intrinsic clock. Here we use directed differentiation of human embryonic stem cells into neuroectoderm to perform a whole-genome CRISPR-Cas9 knockout screen and show that the epigenetic factors Menin and SUZ12 modulate the speed of PAX6 expression during neural differentiation. Genetic and pharmacological loss-of-function of Menin or SUZ12 accelerate cell fate acquisition by shifting the balance of H3K4me3 and H3K27me3 at bivalent promoters, thereby priming key developmental genes for faster activation upon differentiation. We further reveal a synergistic interaction of Menin and SUZ12 in modulating differentiation speed. The acceleration effects were observed in definitive endoderm, cardiomyocyte and neuronal differentiation paradigms, pointing to chromatin bivalency as a general driver of timing across germ layers and developmental stages.

## Abstract (web summary)

Xu et al. perform a whole-genome CRISPR-Cas9 knockout screen using directed differentiation systems. They find that MEN1 and SUZ12 modulate human developmental timing by epigenetically controlling bivalent promoters of developmental genes.

## Main

Embryonic development is characterized by a series of events unfolding in a particular order and pace. While the order is evolutionarily conserved across mammalian species, the rate at which it proceeds varies. The slower pace of human development, compared with most other species, is evident from pre-implantation stage^1^, persists through embryogenesis^2,3^ and becomes progressively exaggerated during maturation^1,3–5^. This species-specific developmental timing is thought to be integral to the determination of organism size, lifespan and tissue complexity^2,6–8^.

In vitro differentiation of stem cells recapitulates the in vivo timing of many developmental processes^9,10^ and mirrors species differences^11–13^, suggesting the existence of an intrinsic clock for cells to track time. Recent studies using comparative stem cell-based in vitro models have identified biochemical reaction rates^14,15^ and metabolic activity^16,17^ as candidate factors contributing to species-specific developmental timing. In parallel, studies conducted primarily in either human or mouse systems have highlighted a role of the epigenetic landscape^18–21^. Notably, variations in the speed of early embryogenesis are predictive of their later developmental features, such as gestation length, brain and body size, age of sexual maturity, and lifespan^7^, raising the possibility that timing mechanisms during embryogenesis underlie a general scaling law that sets the pace of diverse developmental events.

**Fig. 1:**
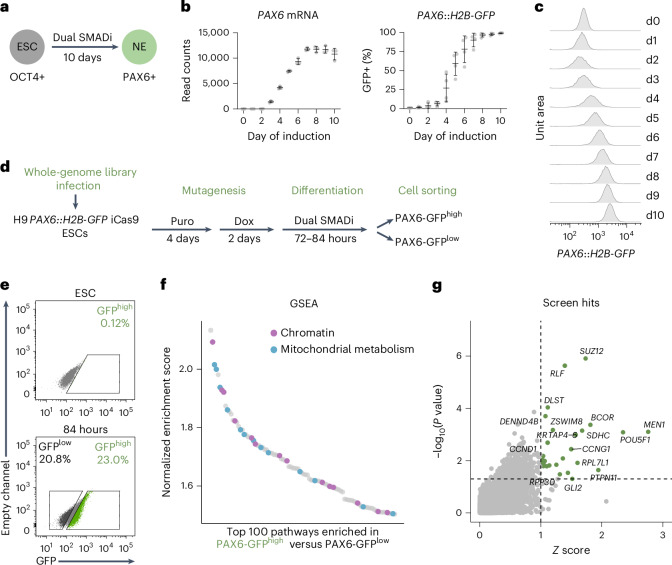
Genome-wide KO screens identify regulators of human neuroectoderm differentiation speed. **a**, Schematic of the neuroectoderm differentiation. NE, neuroectoderm. Dual SMADi, inhibition of SMAD signalling by co-treatment of SB431542 and LDN193189. **b**, Temporal expression of *PAX6* RNA from bulk RNA-seq experiment (left, *n* = 3 independent differentiations) and quantification of GFP expression in the *PAX6::H2B-GFP* cell line from flow cytometry analysis (right, *n* = 4 independent differentiations) during neuroectoderm differentiation. Data are the mean ± s.d. **c**, Representative histogram plots for live GFP expression in the *PAX6::H2B-GFP* cell line undergoing neuroectoderm differentiation. d0, day 0 of induction. **d**, Schematic of the whole-genome CRISPR screen. **e**, Representative flow cytometry gating strategy to isolate PAX6-GFP^high^ and PAX6-GFP^low^ populations. **f**, Waterfall plot of the top 100 GSEA enriched pathways from the ranked gene list, ordered by *Z*-score of PAX6-GFP^high^ versus PAX6-GFP^low^ comparison (top ranks correspond to highly enriched genes in PAX6-GFP^high^ population). Chromatin-related and mitochondrial metabolism-related pathways are highlighted in coloured dots. **g**, Scatter plot of the screen hits (*P-*value < 0.05; *Z*-score > 1) highlighted in green dots. *P-*values were calculated from edgeR’s exact test. Source data

In this study, we conducted an unbiased, whole-genome CRISPR-Cas9 knockout (KO) screen to search for timing regulators during human neuroectoderm differentiation. Neural induction marks the initial step of nervous system development and can be modelled in vitro by the neuroectoderm differentiation of human embryonic stem cells (hESCs) via dual SMAD inhibition^22,23^. Guided by a continuous and uniform induction signal, this directed differentiation captures a well-defined developmental time window that can be tracked by a progressive increase in the neural marker PAX6 expression with a nearly 100% conversion efficiency (Fig. 1a–c). Moreover, the speed of human neuroectoderm differentiation in vitro is similar to the speed of in vivo neural induction where species differences are already apparent^24^. Thus, the transition from hESC to neuroectoderm serves as a proxy for early developmental events and an efficient screening platform for timing regulators.

Using our screening approach, we found that the loss-of-function of two epigenetic factors, Menin (encoded by *MEN1*) and SUZ12, accelerates neuroectoderm differentiation. To address whether they act as a general timing mechanism beyond neural induction, we explored their functions along two additional dimensions: across germ layer lineages (definitive endoderm and cardiac mesoderm), and during a later developmental stage (neurogenesis). These studies define Menin and SUZ12 as modulators of developmental pace that act synergistically across lineages and developmental stages and point to chromatin bivalency as a key regulator of human developmental timing.

## Results

### Genome-wide KO screens identify regulators of human neuroectoderm differentiation speed

**Extended Data Fig. 1:**
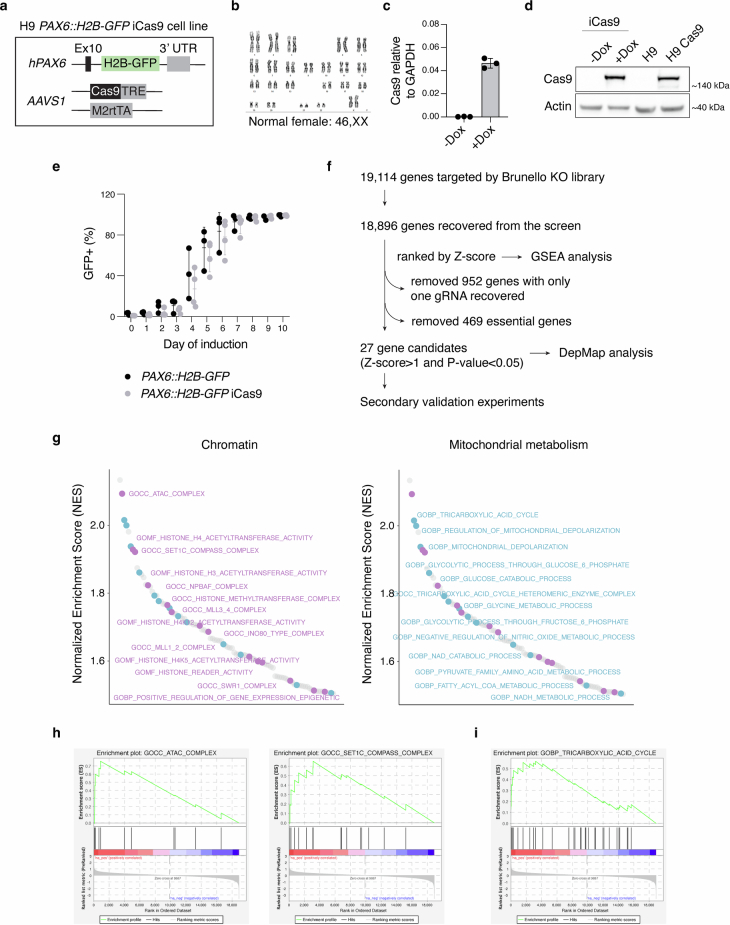
Genome-wide KO screen and analysis. **a**, Schematic of *PAX6::H2B-GFP* iCas9 cell line. iCas9 was inserted at the *AAVS1* locus through TALEN-mediated gene targeting in the *PAX6::H2B-GFP* cell line. TRE, tetracycline response element; M2rtTA, reverse tetracycline transactivator sequence and protein. **b**, Karyotypic analysis of the *PAX6::H2B-GFP* iCas9 H9 hESC clonal cell line used for the study. **c**, Expression of *Cas9* RNA in the *PAX6::H2B-GFP* iCas9 cell line after one day of doxycycline (dox) treatment compared to no dox treatment (n = 3 independent experiments). Data are the mean ± s.d. **d**, Representative Western Blot analysis for Cas9 protein in the *PAX6::H2B-GFP* iCas9 cell line (iCas9) after two days of dox treatment compared to no dox treatment. H9 and H9 carrying a constitutive Cas9 expression (H9 Cas9) were used as negative and positive control respectively. **e**, Flow cytometry analysis of GFP expression during neuroectoderm differentiation in iCas9 cell line and its parental line (n = 4 independent experiments for iCas9 line, n = 3 independent experiments for the parental line). Data are mean ± s.d. **f**, Workflow of screen analysis. **g**, Waterfall plots of the top 100 GSEA enriched pathways from the ranked gene list. Chromatin-related and mitochondrial metabolism-related terms are highlighted in separate plots. **h**, GSEA plots for two of the chromatin-related GO terms, ATAC complex and SET1C/COMPASS complex, that positively correlate with PAX6-GFP^high^ population. **i**, GSEA plot for one of the mitochondrial metabolism-related GO terms, Tricarboxylic acid cycle, that positively correlate with PAX6-GFP^high^ population. Source data

To screen for PAX6 expression in live cells, we first integrated an inducible *Cas9* gene, which expresses Cas9 following doxycycline treatment, into the *AAVS1* locus in the H9 *PAX6::H2B-GFP* hESC reporter cell line^23,25^ (Extended Data Fig. 1a,b). Cas9 protein can be efficiently induced within two days upon doxycycline treatment, and the iCas9 integration does not affect PAX6 expression dynamics (Extended Data Fig. 1c–e). To screen for regulators of differentiation speed at a whole-genome scale, we infected *PAX6::H2B-GFP* iCas9 hESCs with a genome-scale, human CRISPR KO Brunello lentiviral pooled library^26^. hESCs were selected for stable gRNA integration with puromycin, and mutations were induced with doxycycline. We then started neuroectoderm differentiation using the dual SMADi method (Fig. 1d). Given that *PAX6* expression starts to rapidly increase around day 3 to 4 (Fig. 1b and Extended Data Fig. 1e), and thus the acceleration effect of a mutation, if any, would be most pronounced during this time window, we isolated PAX6-GFP^high^ and PAX6-GFP^low^ cells using fluorescence-activated cell sorting (FACS) at the timepoint when we observed the PAX6 level started to increase (Fig. 1e). One replicate was collected at 72 hours (3 days), and two replicates were collected at 84 hours (3.5 days). The gRNA representation in each population was determined by next-generation sequencing. We reasoned that if a gene KO accelerates the differentiation, the gRNA should be repeatedly enriched in the PAX6-GFP^high^ population and depleted in the PAX6-GFP^low^ population. By contrast, if a gene KO has no effect on speed, the gRNA should be randomly distributed between the two populations.

**Extended Data Fig. 2:**
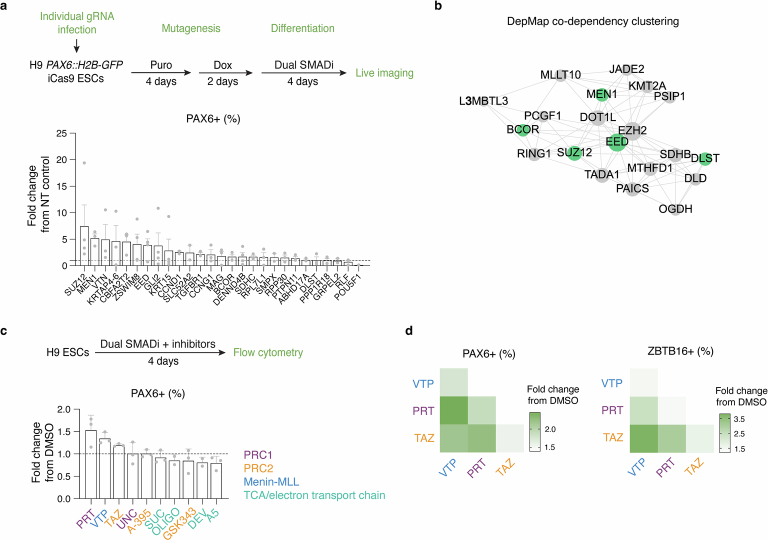
Secondary validation experiments of the screen highlight *MEN1* and *SUZ12* as candidate hits. **a**, Schematic of the genetic approach of the secondary validation experiments and the results of PAX6+ (%) at day 4 normalized to NT control (n = 2 independent differentiations for *SLC52A2*, *SMPX*, *GRPEL2*; n = 4 for *SUZ12*, *ZSWIM8*, *EED*, *GLI2*, *KRT15*, *DENND4B*, *PPP1R18*; n = 3 for the rest). Data are the mean ± s.e.m. **b**, DepMap analysis on the top ranked hits was performed as described in the ‘DepMap analysis’ method section. Gene cluster that includes the most screen hits was shown. **c**, Schematic of the pharmacological approach of the secondary validation experiments and the results of PAX6+ (%) at day 4 normalized to NT control (n = 2 for Oligo and Dev; n = 3 for the rest). Small molecule inhibitors are coloured according to their respective targeting pathways. Details on the inhibitors and their doses used can be found in Supplementary Table 3. Data are the mean ± s.e.m. **d**, Expression of neural markers (PAX6 and ZBTB16) in drug combinations versus DMSO vehicle control at day 4 of neuroectoderm differentiation, assessed by flow cytometry. VTP, VTP50469 (Menin-MLL inhibitor); PRT, PRT4165 (PRC1 inhibitor); TAZ, Tazemetostat (PRC2 inhibitor). Source data

The analysis workflow is illustrated in Extended Data Fig. 1f. First, we compared gRNAs enriched in PAX6-GFP^high^ versus PAX6-GFP^low^ populations and ranked the genes by *Z*-score^27^. Gene-set enrichment analysis (GSEA)^28^ on the ranked gene list showed an enrichment for multiple chromatin remodelling complexes, such as ATAC, SET1C, npBAF and MLL complexes (Fig. 1f and Extended Data Fig. 1g,h), as well as for pathways associated with mitochondrial metabolism, particularly the tricarboxylic acid (TCA) cycle (Fig. 1f, Extended Data Fig. 1g,i and Supplementary Table 1). This is in line with previous studies highlighting both the chromatin landscape and metabolic activity as key regulators of developmental dynamics^29,30^. We then calculated *Z*-score and *P-*value using edgeR^31^ for each gene and defined 27 screen hits (*Z*-score > 1, *P* < 0.05) (Fig. 1g and Supplementary Table 2). To validate the screen hits and gain insights into the molecular pathways that affect differentiation speed, we adopted both genetic and pharmacological approaches. For the genetic validations, we followed up on the screen hits by infecting *PAX6::H2B-GFP* iCas9 hESCs with individual lentiviral gRNA targeting each candidate gene, followed by independent differentiation assays. PAX6 level was assessed by GFP live imaging. Among the 27 screen hits, *SUZ12* and *MEN1* KOs showed the highest fold change of PAX6 expression compared with non-targeting (NT) control (Extended Data Fig. 2a). For the pharmacological approaches, we performed co-essentiality mapping using DepMap database^32,33^ and clustered the screen hits based on their essentiality score correlation. The co-essentiality mapping allowed us to infer functional pathways shared by a subset of the screen hits. One prominent cluster emerged from the analysis that contained 5 out of 27 candidates, including members of Polycomb Repressive Complex (PRC) 1 and 2 (*BCOR; SUZ12, EED*), TCA cycle (*DLST*), and Menin–MLL complex (*MEN1*) (Extended Data Fig. 2b). Accordingly, we selected small molecule inhibitors targeting each pathway or interaction and treated hESCs undergoing differentiation (Supplementary Table 3). PAX6 level was assessed at day 4 by flow cytometry. Of all the pathways perturbed, inhibition of PRC1, PRC2 and Menin–MLL interaction showed the highest fold change of PAX6 expression compared to DMSO-treated cells (Extended Data Fig. 2c). Pairwise combinations of these three perturbations led to an additive effect on accelerating neural markers PAX6 and ZBTB16 (Extended Data Fig. 2d).

Taken together, genetic and pharmacological secondary validation experiments underscored *MEN1* and *SUZ12* as promising candidates for regulating the timing of neuroectoderm differentiation. Intriguingly, Menin and SUZ12 are postulated to have opposing functions on gene expression, namely, Menin/MLL1-mediated histone 3 lysine 4 trimethylation (H3K4me3), an active chromatin mark^34,35^, and PRC2-mediated histone 3 lysine 27 trimethylation (H3K27me3), a repressive chromatin mark^36–38^, yet both KOs led to accelerated *PAX6* expression. Based on these results, we focused on *MEN1* and *SUZ12* for further investigation.

### Menin or SUZ12 inhibition accelerates expression of neuroectodermal genes

**Fig. 2:**
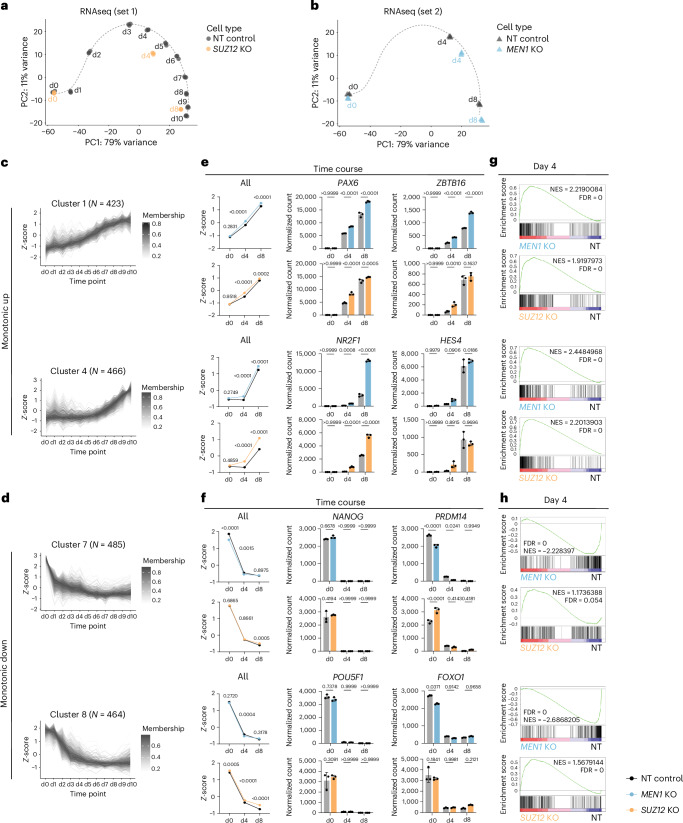
Menin or SUZ12 inhibition accelerates expression of neuroectodermal genes. **a**,**b**, PCA plots of the first set of RNA-seq experiment of NT and *SUZ12* KO samples (**a**) and the second set of RNA-seq experiment of NT and *MEN1* KO samples (**b**). Samples are distributed according to their day of differentiation based on top 500 differentially expressed transcript with variance stabilized normalization. **c**,**d**, Temporal expression patterns of monotonic up clusters 1 and 4 (**c**) and monotonic down clusters 7 and 8 (**d**) in the NT samples identified by TCseq. **e**,**f**, *Z*-score normalized expression of cluster 1 and 4 genes (**e**) and cluster 7 and 8 genes (**f**) in *MEN1* or *SUZ12* KO versus NT control are shown in the line graphs (*n*, number of genes in each cluster; **c**,**d**). Data are the mean ± s.e.m. Normalized counts of selected representative markers of cluster 1 and 4 (**c**) and cluster 7 and 8 (**d**) are shown in the bar graphs (*n* = 3 independent differentiations). Data are the mean ± s.d. *P*-values were calculated from two-way ANOVA test with post-hoc Tukey’s multiple comparisons test. **g**,**h**, GSEA enrichment plots of cluster 1 and 4 genes (**g**) and cluster 7 and 8 genes (**h**) in *MEN1* or *SUZ12* KO versus NT comparisons at day 4. FDR, false discovery rate; NES, normalized enrichment score. Source data

**Extended Data Fig. 3:**
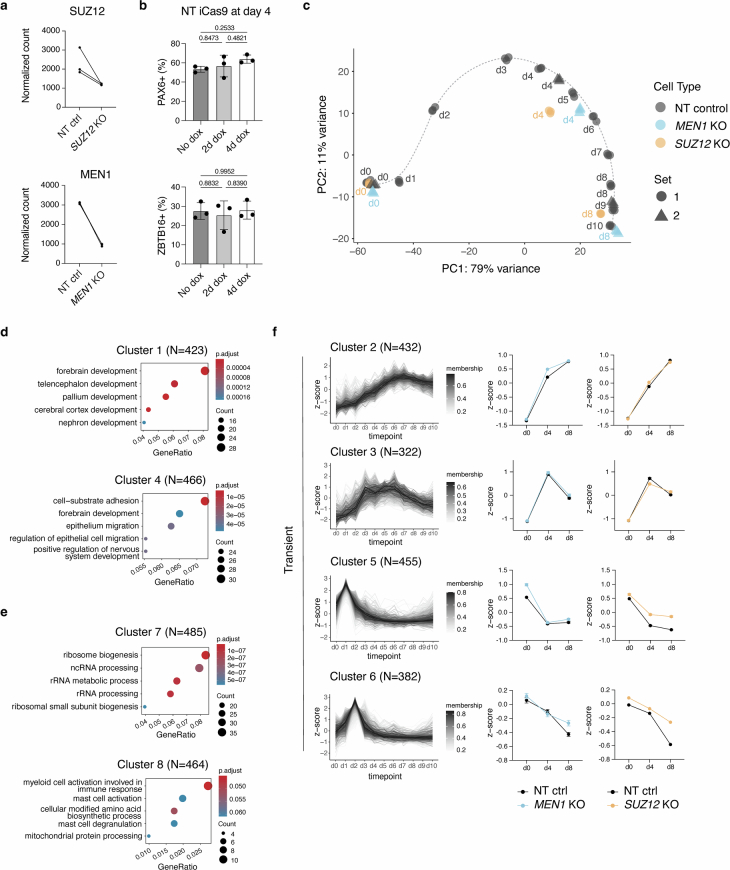
Menin and SUZ12 inhibition largely preserves the broad gene expression patterns. **a**, Normalized counts of *SUZ12* and *MEN1* mRNA in *SUZ12* KO and *MEN1* KO ESCs used in the RNA-seq. **b**, Quantification of PAX6+ and ZBTB16+ cell percentage at day 4 of neuroectoderm differentiation with various durations of doxycycline treatment in NT control, assessed by flow cytometry (n = 3 independent experiments). Data are the mean ± s.d. One-way ANOVA test with post-hoc Tukey’s multiple comparisons test. ns, not significant (*P* > 0.05). **c**, The combined PCA plot. Samples from both sets of RNA-seq experiments were pooled and analysed together to generate the combined PCA plot, which is then divided into two PCA plots by set (Fig. 2a, b). **d**, Gene ontology (GO) analysis of monotonic up clusters 1 and 4. Top five GO terms are plotted. **e**, GO analysis of monotonic down clusters 7 and 8. Top five GO terms are plotted. *P* values for GO analysis were calculated using Fisher’s exact test and adjusted for multiple comparisons using the Benjamini–Hochberg method. **f**, Gene clusters of transient expression from TCseq analysis and *Z*-score normalized expression in *MEN1* or *SUZ12* KO versus NT. Source data

Given that the *PAX6* locus is a known PRC2 target^39^, we first assessed whether the observed effect of *MEN1* and *SUZ12* loss-of-function extends beyond *PAX6* alone and impacts broader transcriptional programmes. Using the same KO strategy as in the CRISPR screen, we infected hESCs with *MEN1*, *SUZ12*, or NT control gRNA. Each cell line underwent the same antibiotic selection and doxycycline treatment to induce mutation. We then differentiated each line individually and performed bulk RNA-seq. We first established a natural (unperturbed) transcriptional trajectory of neuroectoderm differentiation at high temporal resolution by sampling NT cells daily throughout 10-day differentiation. In parallel, we collected *SUZ12* KO cells at day 0, 4, and 8 and mapped their transcriptional profiles onto the natural differentiation trajectory. Principal Component Analysis (PCA) revealed that *SUZ12* KO samples clustered well with NT samples at the hESC stage but showed acceleration upon differentiation at day 4 and 8, with a slight deviation from the unperturbed course (Fig. 2a; Extended Data Fig. 3a). For *MEN1* KO, we repeated the NT and *MEN1* gRNA infection with extended doxycycline treatment (from 2 to 4 days) to improve KO efficiency. This increase in the duration of doxycycline treatment appears to have minimal effect on neural marker expression (Extended Data Fig. 3b). In this second set of differentiations, we collected stage-matched NT and *MEN1* KO cells at days 0, 4 and 8. *MEN1* KO cells also achieved acceleration at day 4 and 8 but exhibited less deviation (Fig. 2b; Extended Data Fig. 3a). PCA was performed with all samples together and the corresponding combined PCA plot is shown in Extended Data Fig. 3c.

**Extended Data Fig. 4:**
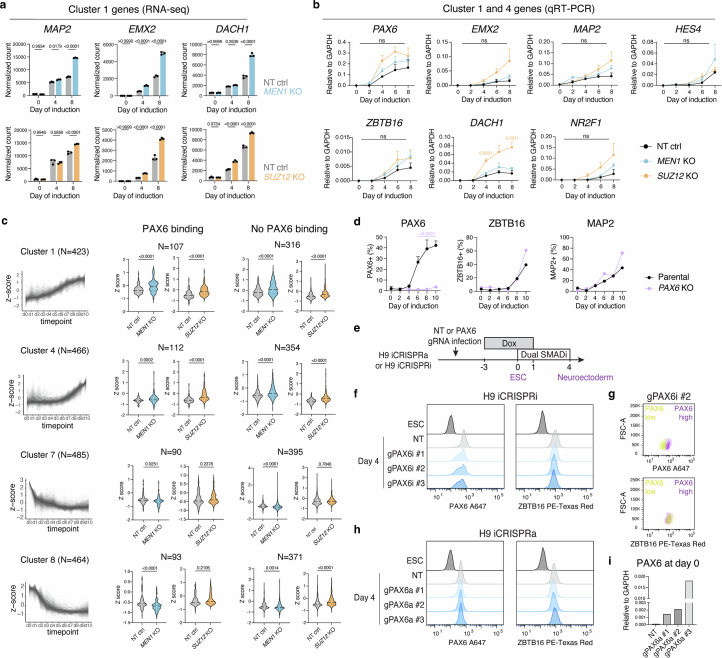
*MEN1* and *SUZ12* KO accelerates gene upregulation during neuroectoderm differentiation. **a**, Normalized counts of additional representative markers of monotonic up cluster 1 (n = 3 independent differentiations). Data are the mean ± s.d. *P* values were calculated from two-way ANOVA test with post-hoc Tukey’s multiple comparisons test. **b**, Time course analysis of mRNA expression of representative markers from monotonic up cluster 1 and 4, relative to housekeeping gene, *GAPDH*, in NT and KO cells, assessed by qRT-PCR (n = 3 independent experiments). Data are the mean ± s.e.m. *P* values were calculated from two-way ANOVA test with post-hoc Dunnett’s multiple comparisons test. **c**, Day 4 expression levels of monotonic up and down genes, grouped by presence or absence of PAX6 binding at the promoter region. A list of PAX6-binding genes in hESCs^41^ (N = 3984) was intersected with each cluster; overlapping genes were classified as ‘PAX6 binding’, while the remaining genes were classified as ‘No PAX6 binding’. *P* values were calculated using two-tailed unpaired *t*-test. **d**, Time course analysis of neural markers PAX6, ZBTB16, and MAP2 in the *PAX6* KO and its parental control lines during neuroectoderm differentiation (n = 4 for PAX6; n = 2 for ZBTB16 and MAP2), assessed by flow cytometry. Data are the mean ± range for PAX6. Data are the mean for ZBTB16 and MAP2. *P* values for PAX6 were calculated from two-way ANOVA test with post-hoc Šidák’s multiple comparisons test. **e**, Schematic of dox-inducible CRISPRa and CRISPRi of PAX6. H9 iCRISPRa or iCRISPRi hESCs were infected with NT or PAX6 gRNA and selected with antibiotics. PAX6 activation or inhibition was induced with 4-day doxycycline treatment (3 days at hESC stage and 1 day during the first day of differentiation). Cells were differentiated into neuroectoderm for 4 days and neural marker expression was assessed by flow cytometry. **f**, Representative histogram plots for neuroectoderm markers PAX6 and ZBTB16 in the H9 iCRISPRi lines. **g**, Representative dot plots of one gPAX6i line at day 4. Cell populations were colour-coded based on their PAX6 expression levels. **h**, Representative histogram plots for neuroectoderm markers PAX6 and ZBTB16 in the H9 iCRISPRa lines. **i**, mRNA expression of *PAX6* relative to housekeeping gene, *GAPDH*, assessed by qRT-PCR, at day 0. ns, not significant (*P* > 0.05). Source data

Based on this observation, we further examined the specificity and extent of transcriptional changes in *MEN1* and *SUZ12* KO cells. We first assessed dynamic patterns of transcriptional changes during the natural differentiation by performing TCseq^40^ using the temporal data of NT cells. This time-course analysis revealed eight distinct gene expression patterns (clusters) underlying neuroectoderm differentiation. Genes in cluster 1 and 4 showed a monotonic upregulated expression and were strongly enriched for forebrain development (Fig. 2c and Extended Data Fig. 3d). Genes in cluster 7 and 8 showed monotonic downregulated expression and were linked to ribosome and mitochondria-related cellular processes (Fig. 2d and Extended Data Fig. 3e). The remaining clusters showed transient expression patterns, with genes peaking at various timepoints during differentiation (Extended Data Fig. 3f). We then mapped the expression level of genes in each cluster in *MEN1* and *SUZ12* KO cells side-by-side with NT controls (Fig. 2e,f and Extended Data Fig. 4a). Both KOs accelerated the upregulation of cluster 1 and 4 genes at day 4 (Fig. 2g), whereas only *MEN1* KO was also able to promote downregulation of cluster 7 and 8 genes (Fig. 2h). qRT-PCR analysis using a finer time course showed a similar trend to the RNA-seq data, indicating faster upregulation of neural genes in the KOs (Extended Data Fig. 4b). The degree of acceleration was similar between PAX6-bound^41^ versus non-PAX6-bound genes (Extended Data Fig. 4c), and PAX6 loss or gain of function did not alter the expression dynamics of other neural markers (Extended Data Fig. 4d–i).

Together, these findings indicate that loss of *MEN1* or *SUZ12* accelerates the progression of gene regulatory networks driving neuroectoderm differentiation, mostly through accelerating upregulation of forebrain development-associated genes. In the cases of monotonic down or transient expressions, we observed a reduced ability of *SUZ12* KO to downregulate gene expression, but the directionality of changes and the overall patterns were preserved (Fig. 2f and Extended Data Fig. 3f).

### Menin and SUZ12 maintain bivalency balance on developmental genes

**Fig. 3:**
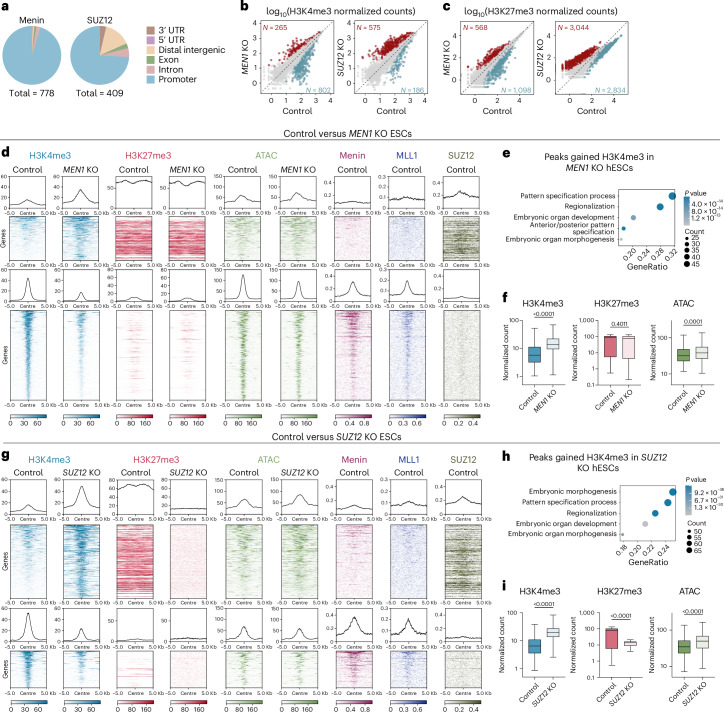
Menin and SUZ12 maintain bivalency balance on developmental genes. **a**, Pie charts of Menin and SUZ12 peaks in the H9 control hESCs mapped to 3′UTR, 5′UTR, distal intergenic, exon, introns and promoter regions. **b**,**c**, Differential analysis for binding of H3K4me3 (**b**) and H3K27me3 (**c**) counts. Each dot represents one H3K4me3 or H3K27me3 peak. Peaks that are significantly enriched (red, *P*_adj_ < 0.05, log_2_FC > 0) and peaks that are significantly depleted (blue, *P*_adj_ < 0.05, log_2_FC < 0) in the KO hESCs, compared to H9 control, are indicated. **d**, Heatmaps show genomic regions with increased (top panel, each row corresponds to a red dot in **b** left panel) and decreased (bottom panel, each row corresponds to a blue dot in **b** left panel) H3K4me3 level in *MEN1* KO hESCs compared with control hESCs. NT hESCs were used as control for ATAC-seq, and unmodified H9 hESCs were used as control for CUT&RUN. The genomic regions are ordered according to the H3K4me3 level in the control sample. **e**, GO analysis on the genes annotated to the peaks with increased H3K4me3 level in *MEN1* KO hESCs. Top five GO terms are shown in the dot plot. **f**, Normalized counts for H3K4me3, H3K27me3 and ATAC peaks for the 265 regions with increased H3K4me3 level in *MEN1* KO hESCs. **g**, Heatmaps show genomic regions with increased (top panel, each row corresponds to a red dot in **b** right panel) and decreased (bottom panel, each row corresponds to a blue dot in **b** right panel) H3K4me3 level in *SUZ12* KO ESCs compared with control hESCs. The genomic regions are ordered according to the H3K4me3 level in the control sample. **h**, GO analysis on the genes annotated to the peaks with increased H3K4me3 level in *SUZ12* KO hESCs. Top five GO terms are shown in the dot plot. **i**, Normalized counts for H3K4me3, H3K27me3 and ATAC peaks for the 575 regions with increased H3K4me3 level in *SUZ12* KO hESCs. **f**,**i**, Box-and-whisker plots: the whiskers represent the minima and maxima, the boxes represent the interquartile range and the centre line represents the median. *P* values were calculated using two-tailed unpaired *t*-test comparing KO to control. Source data

**Extended Data Fig. 5:**
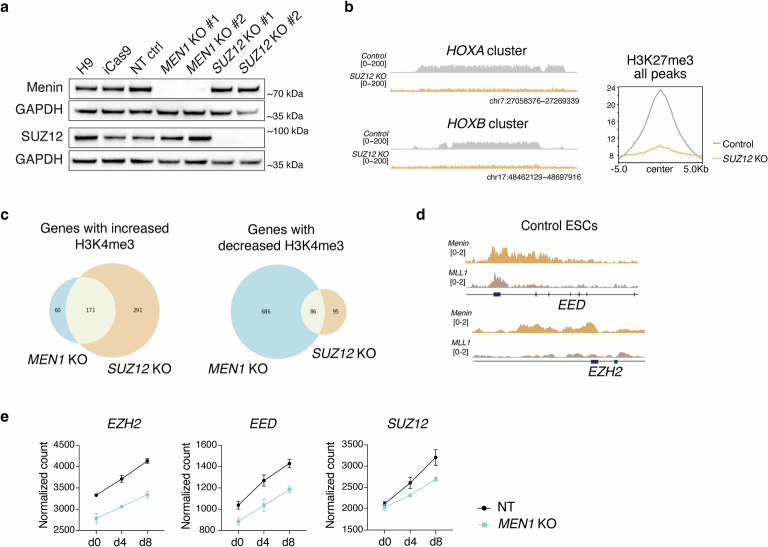
Menin directly binds promoter regions of PRC2 components to regulate their gene expression. **a**, Representative Western Blot analysis for Menin and SUZ12 protein in the clonal *MEN1* KO and *SUZ12* KO cell lines. Two clones for each KO line were used for ATAC-seq. *MEN1* KO #1 line and *SUZ12* KO #1 line were used for CUT&RUN. **b**, Representative track plots of H3K27me3 on *HOXA* and *HOXB* clusters and average profile plot of all H3K27me3 peaks called in NT control and *SUZ12* KO hESCs. **c**, Venn diagrams show overlap in genes with increased (*P*_adj_ < 0.05, log_2_FC > 0) or decreased (*P*_adj_ < 0.05, log_2_FC < 0) H3K4me3 between *MEN1* KO vs. NT and *SUZ12* KO vs. NT comparisons. **d**, Representative track plots depicting the binding of Menin and MLL1 on *EED* and *EZH2*. **e**, Normalized count of PRC2 components (*EZH2*, *EED*, and *SUZ12*) in NT and *MEN1* KO hESCs from the RNA-seq (n = 3 independent differentiations). Data are the mean ± s.d. Source data

Given the described function of Menin and SUZ12 as chromatin regulators^37,42–45^, we then investigated the effect of *MEN1* and *SUZ12* KO on chromatin accessibility and downstream histone modifications using ATAC-seq^46^ and CUT&RUN^47^. We first generated clonal KO hESC lines and confirmed a complete loss of protein via western blot (Extended Data Fig. 5a). Next, we profiled the chromatin occupancy of Menin, Menin’s binding partner MLL1, SUZ12 and their respective histone marks, H3K4me3 and H3K27me3 in hESCs. Menin binds mostly to promoter regions and Menin loss induces broad changes in H3K4me3 localization (Fig. 3a,b). In line with Menin’s role in promoting H3K4 methylation^44,48^, *MEN1* KO hESCs showed significantly reduced H3K4me3 level at 802 peaks. These regions were characterized by co-binding of both Menin and MLL1, low H3K27me3 level and high chromatin accessibility that are indicative of active genes (Fig. 3d, bottom panel). However, *MEN1* KO also led to increased H3K4me3 in a smaller set of peaks (*N* = 265), characterized by low accessibility, abundant H3K27me3, and SUZ12 binding (Fig. 3d, top panel). These characteristics are typical of bivalent promoters of developmental genes^49,50^, which was further supported by Gene Ontology (GO) analysis on genes annotated to these peaks (Fig. 3e). It is worth noting that gain of H3K4me3 at these peaks in *MEN1* KO hESCs did not noticeably affect H3K27me3 level but nevertheless led to a mild increase in chromatin accessibility (Fig. 3f). On the other hand, *SUZ12* KO led to a global erasure of H3K27me3, accompanied by significant H3K4me3 increase at 575 peaks (Fig. 3b,c and Extended Data Fig. 5b). Similar to the peaks that gained H3K4me3 in *MEN1* KO, these peaks were also associated to developmental genes and decorated with both H3K4me3 and H3K27me3 (Fig. 3g, top panel, and h). H3K27me3 erasure at these sites led to a concomitant increase in H3K4me3 and accessibility (Fig. 3i). Interestingly, we also observed that *SUZ12* KO led to a reduction in H3K4me3 binding at a subset of peaks (*N* = 186), which were characterized by binding of Menin and MLL1, while showing low signal for SUZ12 and H3K27me3 (Fig. 3g, bottom panel). Collectively, these data suggest that Menin and SUZ12 regulate the bivalency balance, that is the relative levels of H3K4me3 to H3K27me3, on developmental genes. Loss of Menin or SUZ12 alters this balance towards an activated chromatin state which could prime genes for a faster transcriptional response to differentiation signals.

**Fig. 4:**
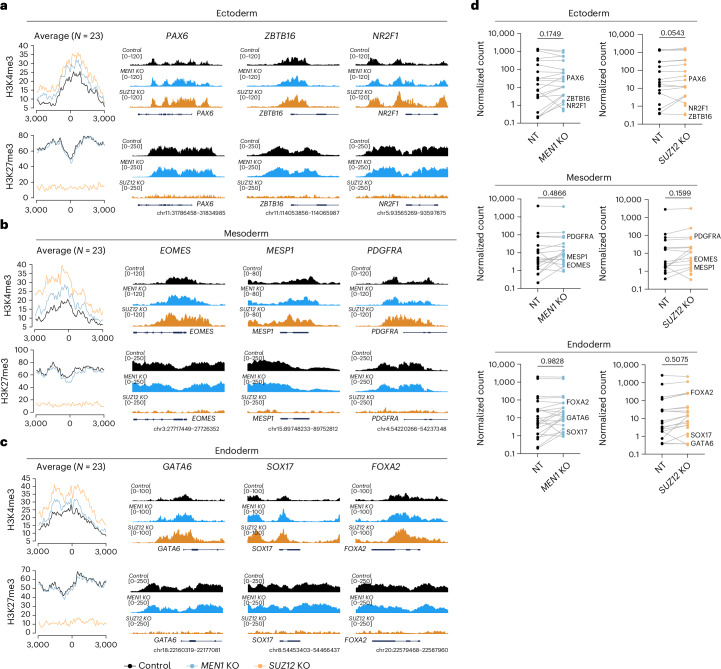
Loss of Menin or SUZ12 shifts the bivalency balance toward activation at lineage genes. **a**–**c**, Average profile plots and example track plots of H3K4me3 and H3K27me3 in control and KO hESCs for ectoderm (**a**), mesoderm (**b**) and endoderm (**c**) lineage markers. For each lineage, the average profile plot of all 23 selected marker genes is shown on the left, with track plots of three representative markers shown on the right. **d**, Normalized expression of lineage genes in NT and KO hESCs from the RNA-seq. Each dot represents one gene. *n* = 23 genes for each lineage. Representative genes for each lineage are labelled. *P* values were calculated using two-tailed paired *t*-test. Source data

**Extended Data Fig. 6:**
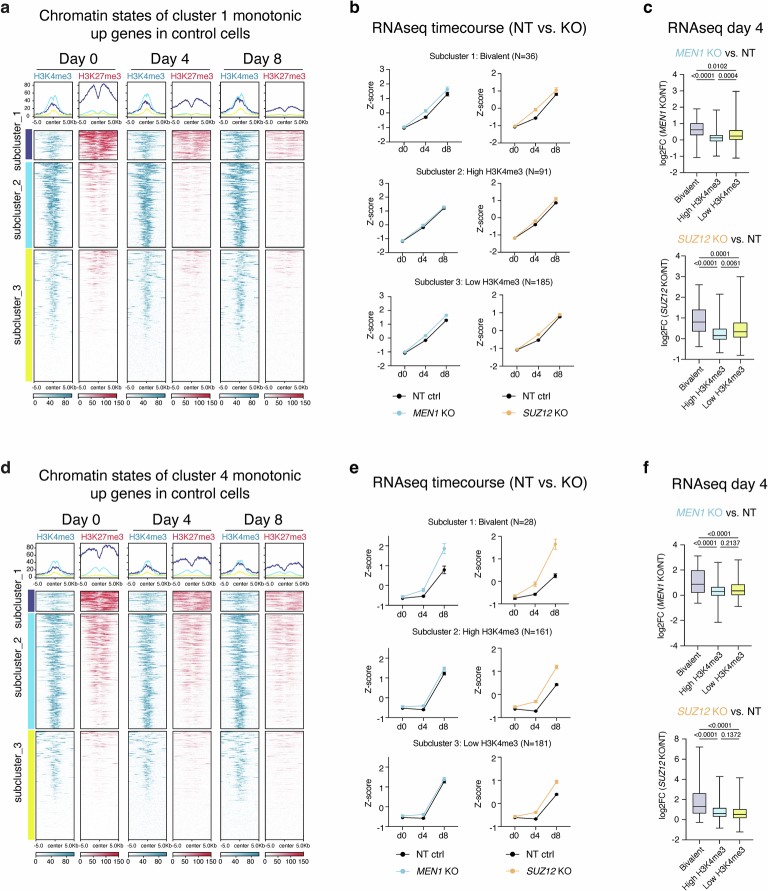
Menin and SUZ12 preferentially accelerate expression of bivalent genes during neuroectoderm differentiation. **a,d**, Unsupervised clustering of H3K4me3 and H3K27me3 peaks on promoters of genes in monotonic up cluster 1 (**a**) and cluster 4 (**d**) identified three chromatin states at hESC stage: bivalent (subcluster_1), high H3K4me3 (subcluster_2), and low H3K4me3 (subcluster_3). **b,e**, *Z*-score normalized expression of genes belonging to each chromatin state in cluster 1 (**b**) and cluster 4 (**e**) in *MEN1* or *SUZ12* KO and NT during neuroectoderm differentiation (n = number of genes in each subcluster, indicated on the graphs). Data are the mean ± s.e.m. **c,f**, Histograms of fold change of gene expression in each chromatin state using normalized count in NT and KOs. Box-and-whisker plots: the whiskers represent the minima and maxima, the boxes represent the interquartile range, and the centre line represents the median (n = number of genes in each subcluster, indicated in **b,e**). *P* values were calculated using one-way ANOVA test with post-hoc Tukey’s multiple comparisons test. Source data

The similarity in the chromatin profile of the peaks with increased H3K4me3 level between *MEN1* and *SUZ12* KOs (bivalent, SUZ12-bound), as well as of the peaks with decreased H3K4me3 level (active, Menin-bound) was further supported by a substantial overlap between the two KOs in both peak groups (Extended Data Fig. 5c), indicating a potential functional interaction between Menin and SUZ12 during H3K4me3 redistribution. Indeed, we observed a direct binding of Menin on *EZH2* and *EED* promoters and a marked reduction of EZH2 and EED transcripts in *MEN1* KO hESCs (Extended Data Fig. 5d,e). These results are compatible with a model in which Menin loss releases H3K4me3 from active genes while alleviating PRC2-mediated repression on bivalent regions, thereby allowing a targeted increase of H3K4me3 on bivalent regions. Consistent with these findings, we observed that despite broadly accelerating gene upregulation during neuroectoderm differentiation, Menin and SUZ12 loss predominantly affected genes with a bivalent chromatin state at the hESC stage, which showed the greatest degree of acceleration compared to other chromatin states (Extended Data Fig. 6a–f). To further explore whether this ‘priming’ effect is lineage-specific, we compiled a list of lineage markers of ectoderm, mesoderm and endoderm^51–53^ (Supplementary Table 4), and examined the bivalency balance at their promoters in *MEN1* and *SUZ12* KO at hESC stage. We found a consistent increase in H3K4me3 across marker genes of all three germ layers in both KOs (Fig. 4a–c). Importantly, this change in bivalency balance alone was not sufficient to induce gene expression at hESC stage (Fig. 4d). These findings indicate that *MEN1* and *SUZ12* KOs trigger chromatin changes that facilitate, rather than causally instruct, gene expression changes and that such facilitation enables accelerated cell fate acquisition.

### Targeting Menin or SUZ12 accelerates differentiations of other lineages and later stages

**Fig. 5:**
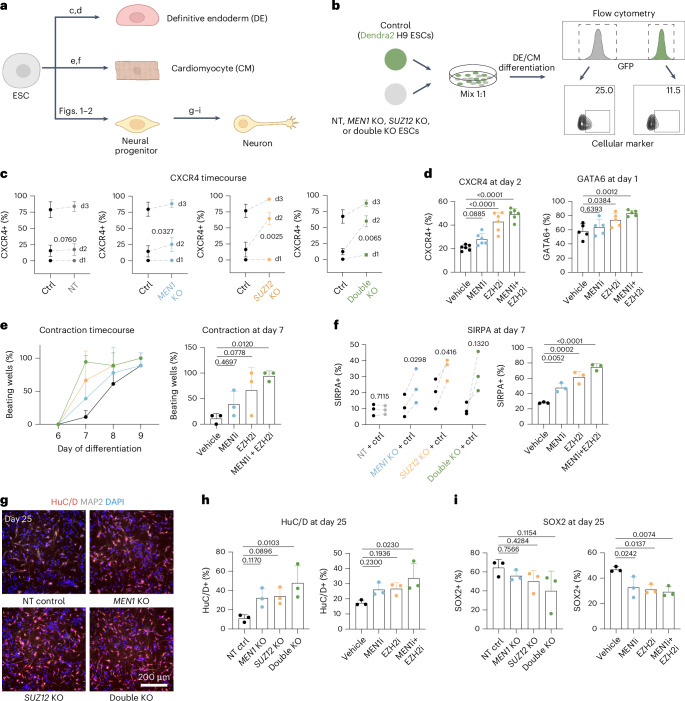
Targeting Menin or SUZ12 accelerates differentiations of other lineages and later stages. **a**, Schematics of differentiation paradigms. Letters on the arrows indicate the corresponding panels. **b**, Schematics of differentiation strategy, applicable to **c** and **f**, to test the KO effects. Briefly, H9 ESCs carry a fluorescent protein, Dendra2, was mixed in a 1:1 ratio with NT or KO ESCs. DE or CM differentiation was performed on mixed cells. During flow cytometry, Dendra2 control cells are separated from NT or KO cells based on GFP signal, and cellular marker expression was assessed in each population. **c**, Time course analysis of CXCR4+ cell percentage in control versus NT or KO cells during DE differentiation (*n* = 3 independent experiments). *P-*values for day 2 comparisons were calculated using two-tailed paired *t*-test. **d**, Quantification of CXCR4+ cell percentage at day 2 (*n* = 6 independent experiments) and GATA6+ cell percentage at day 1 (*n* = 5 independent experiments) of DE differentiation with small molecule inhibitor treatment. *P* values were calculated using one-way ANOVA test with post-hoc Dunnett’s multiple comparisons test. **e**, Analysis of contraction phenotype during CM differentiation with small molecule inhibitor treatment (*n* = 3 independent experiments). *P-*values were calculated using one-way ANOVA test with post-hoc Dunnett’s multiple comparisons test. **f**, Quantification of SIRPA+ cell percentage at day 7 of CM differentiation in NT and KOs (left, *n* = 3 independent experiments), and small molecule inhibitors (right, *n* = 3 independent experiments). *P-*values were calculated using two-tailed paired *t*-test (left) and one-way ANOVA test with post-hoc Dunnett’s multiple comparisons test (right). **g**, Representative images of day 25 cells expressing neuronal markers HuC/D and MAP2. **h**, Quantification of HuC/D+ cell percentage at day 25 of neurogenesis in NT and KOs (left, *n* = 3 independent experiments), and small molecule inhibitors (right, *n* = 3 independent experiments). *P-*values were calculated using one-way ANOVA test with post-hoc Dunnett’s multiple comparisons test. **i**, Quantification of SOX2+ cell percentage at day 25 of neurogenesis in NT and KOs (left, *n* = 3 independent experiments), and small molecule inhibitors (right, *n* = 3 independent experiments). *P-*values were calculated using one-way ANOVA test with post-hoc Dunnett’s multiple comparisons test. All data are the mean ± s.d. *P-*values were indicated on the plots unless not significant (ns, *P* > 0.05). **a** created with BioRender.com. Source data

**Extended Data Fig. 7:**
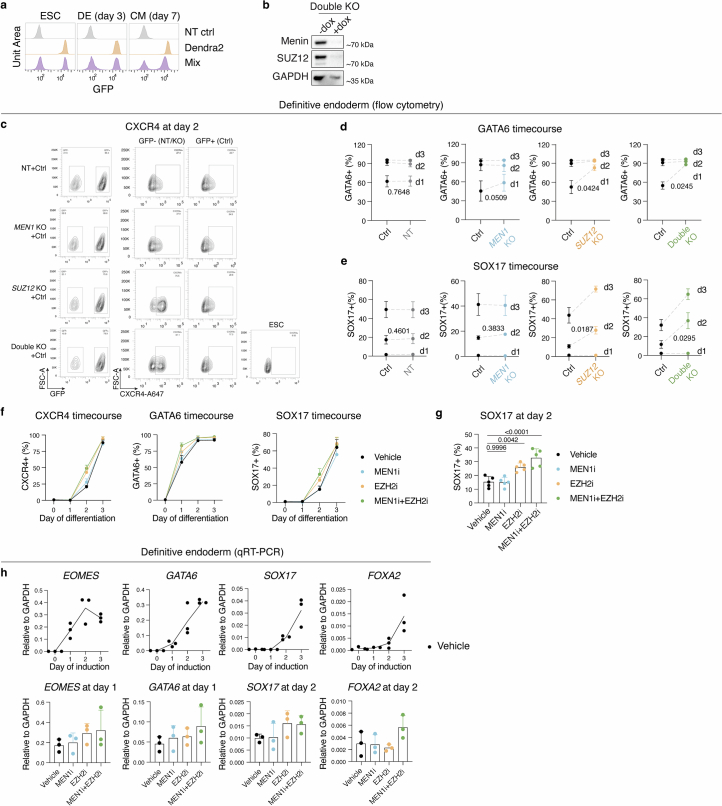
Menin and SUZ12 inhibition accelerates DE differentiation. **a**, Representative flow cytometry histograms show GFP signals in H9 Dendra2, NT, and mix conditions at ESC, DE, and CM stages. **b**, Representative Western Blot analysis to confirm the protein loss in the double KO hESCs. **c**, Representative flow cytometry analysis for CXCR4 expression at day 2 of DE differentiation. Dendra2 control and NT/KO cells were separated by GFP signal, and CXCR4+ gating was established using ESC as the negative control. **d**, Time course analysis of DE marker GATA6+ cell percentage in control versus NT or KO cells during DE differentiation (n = 3 independent experiments). Data are the mean ± s.e.m. *P* values for day 1 comparisons were calculated using two-tailed paired *t*-test. **e**, Time course analysis of DE marker SOX17+ cell percentage in control versus NT or KO cells during DE differentiation (n = 3 independent experiments). *P* values for day 2 comparisons were calculated using two-tailed paired *t*-test. Data are the mean ± s.e.m. **f**, Time course analysis of DE markers CXCR4 (n = 6 independent experiments), GATA6 (n = 5 independent experiments), and SOX17 (n = 5 independent experiments) with small molecule inhibitors. Data are the mean ± s.d. **g**, Quantification of SOX17 expression at day 2 with small molecule inhibitors (n = 5 independent experiments). Data are the mean ± s.d. *P* values were calculated using one-way ANOVA test with post-hoc Dunnett’s multiple comparisons test. ns, not significant (*P* > 0.05). **h**, mRNA expression of representative DE markers, relative to housekeeping gene, *GAPDH*, assessed by qRT-PCR (n = 3 independent differentiations). Upper panels: Time course analysis in the vehicle control cells. Data are each replicate with means connected. Bottom panels: Quantification of DE markers at the onset of gene upregulation following inhibitor treatment. Data are the mean ± s.d. No significant difference among means (*P* < 0.05) for all markers, tested by one-way ANOVA test with post-hoc Dunnett’s multiple comparisons test. Source data

Given the observation that Menin and SUZ12 maintain bivalency balance on developmental genes across germ layers, we hypothesized that they regulate speed of differentiation towards other lineages as well. To test this hypothesis, we assessed the effect of Menin and SUZ12 loss-of-function on hESC differentiations into definitive endoderm (DE) and cardiomyocyte (CM) (Fig. 5a). We chose these two differentiation paradigms as they represent lineages from the two alternative germ layers, can be directed in vitro at high efficiency, and are characterized by the acquisition of a set of well-defined markers^54,55^. In the case of cardiomyocyte, the differentiation assay also allowed us to test, beyond mesoderm specification, the impact of Menin and SUZ12 on a functional readout such as cardiomyocyte contraction. As the robustness of DE and CM differentiation is sensitive to the initial cell density, we mixed NT or KO hESCs with H9 ESCs that carry a green fluorescent protein, Dendra2, at 1:1 ratio before inducing differentiation. The two populations are easily distinguishable by flow cytometry, and each population can be measured individually for marker expression (Fig. 5b and Extended Data Fig. 7a). This setting eliminates the experimental variations due to seeding density and allows us to use H9 Dendra2 ESCs as an internal control for NT and KO cells. To test potential synergistic effect of Menin and SUZ12, we also generated a double KO line (Extended Data Fig. 7b).

During DE differentiation, NT cells showed similar expression dynamics of the definitive endoderm markers GATA6 and CXCR4 (refs. ^54,56^), compared to the H9 Dendra2 control cells. *MEN1*, *SUZ12* and double KO cells showed a marked increase in GATA6 at day 1 and CXCR4 at day 2 (Fig. 5c and Extended Data Fig. 7c,d). In addition to genetic KOs, we explored the functional relevance of Menin–MLL1 interaction and PRC2 by using two inhibitors validated in the neuroectoderm secondary screen (Extended Data Fig. 2c): VTP50469 (ref. ^57^), a Menin–MLL1/2 inhibitor (MEN1i), and Tazemetostat^58^, an EZH2 inhibitor functionally targeting PRC2 (EZH2i), respectively. In line with the genetic KOs, MEN1i and EZH2i treatment during differentiation accelerated expression of the endoderm markers GATA6 and CXCR4 (Fig. 5d and Extended Data Fig. 7f). EZH2i or *SUZ12* KO also promoted SOX17 expression (Extended Data Fig. 7e,g). Notably, the combination of MEN1i and EZH2i resulted in a further increased expression of DE markers. qRT-PCR on DE markers (*EOMES*, *GATA6*, *SOX17* and *FOXA2*) at the onset of gene induction recapitulated the trend of elevated expression in Menin- and SUZ12-targeted cells (Extended Data Fig. 7h).

**Extended Data Fig. 8:**
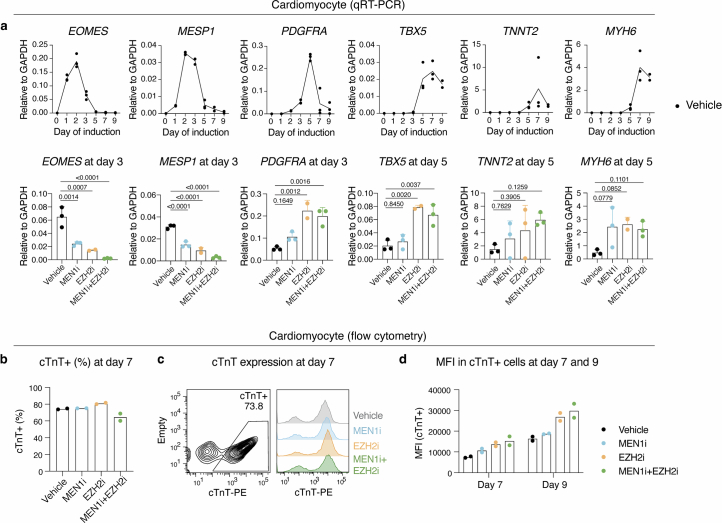
Menin and SUZ12 inhibition accelerates CM differentiation. **a**, mRNA expression of representative CM markers, relative to housekeeping gene, *GAPDH*, assessed by qRT-PCR (n = 3 independent differentiations). Upper panels, Time course analysis in the vehicle control cells. Data are each replicate with means connected. Bottom panels, Quantification of CM markers at the onset of gene upregulation or downregulation following inhibitor treatment. *P* values were calculated using one-way ANOVA test with post-hoc Dunnett’s multiple comparisons test. *P* values were indicated on the plots unless not significant (ns, *P* > 0.05). **b**, Quantification of cTnT+ cell percentage at day 7 (n = 2 independent differentiations). **c**, Representative contour plot and histograms show bimodal distribution of cTnT expression level in day 7 cells, and the gating strategy to define cTnT+ cells. **d**, Mean fluorescent intensity (MFI) of cTnT in cTnT+ cells in day 7 and 9 cells (n = 2 independent differentiations). Source data

For CM differentiation, qRT-PCR confirmed that cells progressed through the following differentiation stages: cardiac mesoderm (*EOMES*, *MESP1*), cardiac progenitor (*PDGFRA*, *TBX5*) and CM (*TNNT2*, *MYH6*)^59^. Cells treated with MEN1i or EZH2i appeared to move more quickly through those stages with a reduction in cardiac mesoderm markers by day 3 and increased expression of cardiac progenitor and CM markers from day 3 onwards (Extended Data Fig. 8a). To assess the functional property of CM, hESCs were seeded in a multi-well plate and treated with MEN1i and/or EZH2i for the duration of differentiation. CM contraction can typically be detected around days 7–9, so we examined the percentage of wells that exhibit contraction during this time window. While we observed contraction in all control wells around day 9, MEN1i or EZH2i treatment induced premature contraction by day 8, and the combined inhibition by day 7 (Fig. 5e). In line with the contraction phenotype, we detected a higher percentage of cells expressing CM-specific cell-surface marker, SIRPA (ref. ^60^), in KOs and drug-treated cells at day 7 (Fig. 5f). While drug treatment did not further increase the percentage of cells expressing cardiac troponin (cTnT, encoded by *TNNT2*) at day 7—which was already around 75%—the treatment did further elevate the cTnT expression level in the cTnT+ population (Extended Data Fig. 8b–d). Combined inhibition further enhanced the premature contraction and increased marker expression observed in single drug inhibition. These results indicate that, in addition to neuroectoderm, Menin and SUZ12 regulate the speed of transition from ESCs to endoderm and a mesoderm-derived lineage as well.

**Extended Data Fig. 9:**
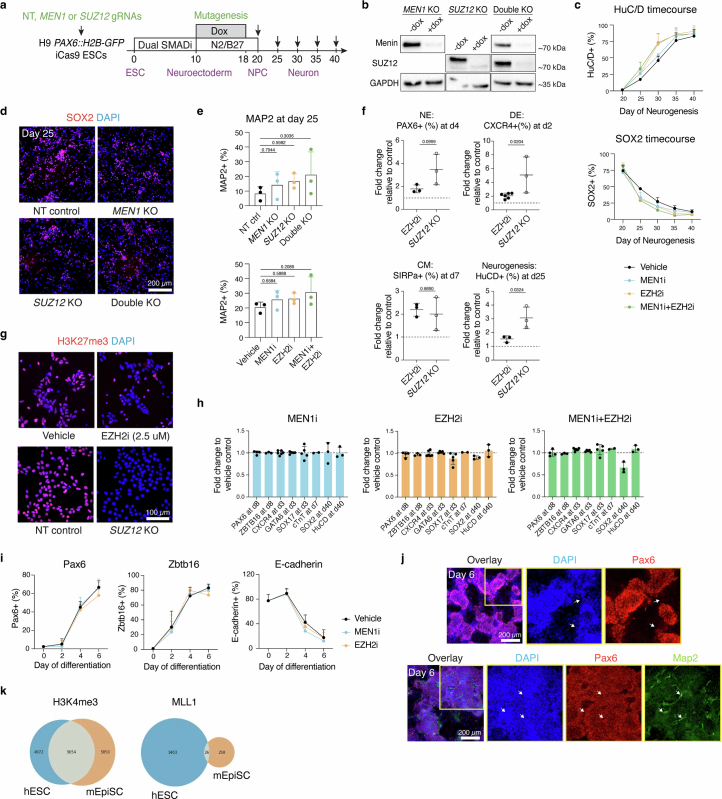
Menin and SUZ12 inhibition accelerates neurogenesis but not mouse neuroectoderm differentiation. **a**, Schematic of dox-inducible KO at neuroectoderm stage. **b**, Representative Western Blot analysis to confirm the protein loss at the NPC (day 20) stage. **c**, Time course analysis of neuronal marker HuC/D and neural progenitor marker SOX2 with small molecule inhibitors (n = 3 independent experiments). **d**, Representative images of day 25 cells expressing neural progenitor marker SOX2. **e**, Quantification of MAP2 expression at day 25 of neurogenesis in small molecule inhibitors (left, n = 3 independent experiments), and NT and KOs (right, n = 3 independent experiments). *P* values were calculated using one-way ANOVA test with post-hoc Dunnett’s multiple comparisons test. **f**, Fold change of representative marker expression relative to control in EZH2i versus *SUZ12* KO (n = 6 for EZH2i in DE; n = 3 for the rest). *P* values were calculated using two-tailed unpaired *t*-test. **g**, Representative images of H3K27me3 in EZH2i versus *SUZ12* KO. **h**, Fold change of representative marker expression at differentiation endpoint with drug treatment (n = 2 for cTnT; n = 3 for ZBTB16, SOX2, HuCD; n = 4 for PAX6; n = 5 for GATA6, SOX17; n = 6 for CXCR4). **i**, Quantification of neural markers, Pax6 and Zbtb16, and a pluripotency marker, E-cadherin, during neuroectoderm differentiation of mouse epiblast stem cells (mEpiSCs), assessed by flow cytometry (n = 3 independent differentiations). No significant difference among means (*P* < 0.05) for all markers, tested by two-way ANOVA test with post-hoc Dunnett’s multiple comparisons test. **j**, Representative images of day 6 mouse cells expressing the neuroectoderm marker Pax6 and the neuronal marker Map2. White arrows point to Pax6-negative non-neural cells (top panels) and Map2-positive neurons (bottom panels). **k**, Venn diagrams show overlap in genes with H3K4me3 or MLL1 binding between hESCs and mEpiSCs. ChIP-seq data in mEpiSCs were downloaded from the ChIP-Atlas database^93^. All data are the mean ± s.d. Source data

Next, we explored whether Menin and SUZ12 can further affect timing at a later developmental stage, independent of lineage specification from ESC stage. We focused on neurogenesis and induced genetic KO only after the differentiating cells have reached neuroectoderm stage. To this end, we infected iCas9 hESCs with gRNAs and did not induce Cas9 until day 10 (Extended Data Fig. 9a,b). For the pharmacological approach, MEN1i or EZH2i was applied starting at the neural progenitor stage (from day 20 onward). We probed neurogenesis speed by assessing the transition from SOX2+ neural progenitor to post-mitotic neuron, marked by HuC/D and MAP2. Both genetic and pharmacological manipulations led to a higher percentage of HuC/D+ or MAP2+ neurons and a further reduction of SOX2+ progenitors at day 25. Double KO or combined MEN1i and EZH2i treatment further enhanced this impact (Fig. 5g–i and Extended Data Fig. 9c–e).

Overall, chemical and genetic inhibition of Menin and SUZ12 produced similar phenotypes in all differentiation paradigms tested, suggesting that Menin–MLL interaction and EZH2–PRC2 play a crucial role in timing regulation across lineages and developmental stages. *SUZ12* KO appears to achieve a higher degree of accelerated gene expression than EZH2i in neuroectoderm, DE and neuronal differentiation, which could be explained by incomplete depletion of H3K27me3 by EZH2i (Extended Data Fig. 9f,g) and potential EZH1-PRC2 activity^61^. In addition, MEN1i or EZH2i did not induce detectable cell fate changes, as evidenced by similar expression levels of markers at the differentiation endpoint compared to control (Extended Data Fig. 9h).

Finally, we explored whether the functions of Menin and SUZ12 are conserved in another species. We differentiated mouse epiblast stem cells (mEpiSCs) into neuroectoderm with MEN1i or EZH2i treatment. The dynamics of Pax6 and Zbtb16 remained largely unaffected, and the downregulation of E-cadherin was only mildly accelerated (Extended Data Fig. 9i). However, we observed both Pax6-negative non-neural cells and Map2-positive neurons in addition to Pax6-positive neuroectoderm, complicating direct timing comparison using mouse cells (Extended Data Fig. 9j). Furthermore, despite the broad overlap of H3K4me3 peaks between hESCs and mEpiSCs, Menin’s binding partner, MLL1, exhibited distinct binding patterns (Extended Data Fig. 9k), raising the possibility that Menin’s functional role differs between mouse and human cells during primed pluripotency.

## Discussion

CRISPR-Cas9 screening and stem cell technologies have enabled the systematic discovery of mechanisms underlying cell fate specifications. Here, we further exploit the potential of these technologies by establishing a genome-wide loss-of-function screen to identify molecular regulators of human neuroectoderm differentiation speed. By focusing on a quantitative cellular marker rather than a binary outcome, we demonstrate the broad utility of pooled genetic screens in not only identifying factors essential for fate specification, but in pinpointing temporal regulators as well.

Our screen highlighted Menin and SUZ12 as key regulators of neural induction speed. Although we focused on epigenetic factors in this study, we also uncovered genes involved in metabolic pathways (for example, *SDHC* and *DLST*), pathways which have been linked to species-specific developmental timing^16,17,62^. However, we were not able to validate these hits using an arrayed format. It is possible that the effects of perturbing metabolic genes are dependent on environmental conditions^63^, which could be different between the pooled format used in the screen and the arrayed format used in the validation. The perturbations may also influence cell growth and proliferation, the negative effect of which could be masked in a pooled setting^64^. Interestingly, we did not uncover genes known to be directly involved in protein synthesis and degradation processes, which have also been linked to timing regulation^14,15^. Given that these cellular pathways are orchestrated by multiprotein complexes, knocking out a single gene may not necessarily generate detectable phenotypic changes^65–67^. Finally, while our whole-genome screens allowed us to search for intrinsic mechanisms in an unbiased manner, we cannot rule out that additional, non-cell autonomous mechanisms might influence timing, although the degree to which non-autonomous factors affect timing remains unclear^24,68^. Future characterizations of the interplay between epigenetic and metabolic factors and how they are influenced by extrinsic factors may provide a more complete understanding of how developmental timing is regulated.

Mutations in PRC2 components have been associated with growth-related disorders, such as Weaver syndrome^69,70^ and Imagawa–Matsumoto syndrome^71^, reflecting PRC2’s broad influence on growth and development. It will be interesting to assess whether the timing differences observed reported in the current study may help in explaining phenotypes characteristic to those neurodevelopmental disorders. On the other hand, Menin is known for its role in multiple endocrine neoplasia and in leukaemia, whereas its function in a normal developmental context remains largely unexplored. In this study, we uncovered a role of Menin in regulating developmental timing. *MEN1* KO triggers H3K4me3 reduction at Menin-binding sites and an unexpected gain of H3K4me3 specifically at developmental genes. The specificity of H3K4me3 relocation to developmental genes could be achieved through MLL1/2 and their binding partners. Recent studies in lymphoblasts and lung adenocarcinoma cells suggested that upon Menin loss, MLL1 drives H3K4me3 relocation towards bivalent promoters and repetitive genomic regions, respectively^72,73^. However, it is still unclear what mediates MLL1 redistribution, a process that appears to be cell-type specific. In fact, MLL1/2 and their binding partners exhibit distinct chromatin localization and functions^74–76^. Thus, comprehensive chromatin profiling will be required to investigate the possibility that Menin competes with other MLL1 binding partners for MLL1 binding and H3K4me3 deposition. A functional interplay between Menin and PRC2 on gene regulation has been described in leukaemia and lymphoma cells^77,78^. Here, we found that Menin directly regulates gene expression of PRC2 components, particularly EZH2 and EED. Reduced expression of PRC2 components upon *MEN1* KO could decrease PRC2 occupancy and alleviate repression on developmental genes, facilitating further H3K4me3 deposition. Taken together, our results support a model in which Menin limits the speed of unfolding developmental programmes via its dual functions, maintaining H3K4me3 on active chromatin and sustaining PRC2 repression on bivalent chromatin.

Our finding that Menin and SUZ12’s functions converge on maintaining H3K4me3 and H3K27me3 balance on developmental gene promoters underscores a key role of chromatin bivalency in timing regulation. Recent studies have identified PRC2-mediated H3K27me3 on bivalent genes as a driver for maturation timing in human cortical neurons^18^ and mouse cerebellar granule cells^20,21^. Our data suggest that chromatin bivalency acts as a rate-limiting mechanism already during early embryonic development. Furthermore, we show that both repressive and activating histone modifications contribute to timing. In particular, increased H3K4me3 alone at bivalent sites upon *MEN1* KO enables faster gene activation during differentiation, potentially through H3K4me3’s role in promoting transcriptional elongation^79^. Further studies to test whether Menin loss also exerts an effect on neuronal maturation and how bivalency is dynamically remodelled from pluripotency to terminally differentiated state across the three germ layers will be important in further testing the general applicability of this model.

While we did not observe similar acceleration effect in the neuroectoderm differentiation of mEpiSCs in this study, the heterogeneity of the differentiated mouse cell population calls for a more robust mouse differentiation protocol to accurately assess whether the proposed mechanism is conserved across species. Nevertheless, the differential binding patterns of MLL1 between hESCs and mEpiSCs suggest that bivalency may be regulated by distinct epigenetic regulators across species.

In summary, our results suggest that Menin and SUZ12’s role in timing regulation is conserved across germ layer specifications and developmental stages, highlighting bivalency balance as a driver for human-specific timing. The functional overlap between Menin and PRC2 and the additive effects of combined inhibition described in this study can be exploited for manipulating developmental time in vitro, with broad applications in stem cell therapy and disease modelling.

## Methods

### hPSC lines and cell culture

All work using human pluripotent stem cells (hPSCs) was approved by and conducted in compliance with the Tri-Institutional Stem Cell Initiative Embryonic Stem Cell Research Oversight Committee (Tri-SCI PSCRO) at Memorial Sloan Kettering Cancer Center, Rockefeller University and Weill Cornell Medicine. hPSC line WA09 (H9; 46XX) was from WiCell Stemcell Bank. The PAX6::H2B-GFP line was generated previously and derived from WA09 hPSCs^23^. The PAX6::H2B-GFP iCas9 line was derived from the PAX6::H2B-GFP line. hPSCs were maintained in Essential 8 media (E8; Life Technologies, #A1517001) in feeder-free conditions on vitronectin-coated plates (VTN-N; Thermo Fisher, #A14700). hPSCs were passaged as clumps every 3–4 days with EDTA (0.5 M EDTA/PBS) and routinely tested for mycoplasma contamination.

### Cell line engineering

PAX6::H2B-GFP iCas9 knock-in hPSCs line was generated using CRISPR/Cas9-mediated homologous recombination by transfecting PAX6::H2B-GFP hPSCs with Hygro-Cas9 donor plasmid and AAVS1-Neo-M2rtTA (Extended Data Fig. 1a). NT, MEN1 KO, SUZ12 KO, and double KO hPSC lines were generated by transfecting PAX6::H2B-GFP iCas9 hPSCs with the following gRNAs: NT, AGCGCAGATAGCGCGTATCA; MEN1, CCAGGCATGATCCTCAGACA; SUZ12, TTCTCTATAACAACAATACA. For RNA-seq, polyclonal populations with stably integrated gRNAs were selected using puromycin and underwent doxycycline-induced mutations. For ATAC-seq, CUT&RUN and lineage differentiation assays, selected clones with gRNA integration were validated by genomic PCR and screened for Karyotype banding. To minimize variability due to long-term culture of edited cells, mutations were induced before each differentiation assay by 2-day treatment of 1 μg ml^−1^ doxycycline. Protein loss was confirmed by western blot. H9 Dendra2 line was generated by transfecting H9 hPSCs with Dendra2-Neo donor plasmid. Selected clones were validated by flow cytometry.

### Neural differentiation

To induce neuroectodermal fate, hESCs were dissociated into single cells using Accutase (Innovative Cell Technologies, #AT104-500) and plated at 300,000 cells per cm^2^ onto Matrigel-coated wells (Corning, #354234) in E8 medium, supplemented with 10 μM Y-27632 (R&D, #1254) (day −1). On the following day (day 0), culture medium was replaced with Essential 6 medium (E6; Thermo Fisher Scientific, #A1516401) supplemented with 10 μM SB431542 (Tocris, #1614) and 100 nM LDN193189 (Stem Cell Technologies, #72142). Differentiation medium was changed daily from day 1 to 9.

Generation of neurons continues as following. From day 10 to 20, cells were cultured in 1:1 Neurobasal (Thermo Fisher, #21103049) and DMEM/F12 (Thermo Fisher, #11330-032) medium supplemented with 1X N2 (Thermo Fisher, #17502048), 1X B27 without vitamin A (Thermo Fisher, #12587010) and Penicillin Streptomycin (Pen/Strep; Thermo Fisher Scientific, #15140-122) to generate a neurogenic population of cortical NPCs. On day 20, cells were either cryopreserved in STEM-CELLBANKER freezing media (Amsbio #11924) at approximately ten million cells per vial, or induced for neurogenesis as following: NPCs were dissociated using Accutase for 30 min and replated at a density of 120,000 cells per cm^2^ in plates coated with poly-l-ornithine (PO; Sigma-Aldrich, #P3655), laminin (LAM; R&D, #3400-010-1) and fibronectin (FN; Thermo Fisher, #356008). After replating, cells were cultured in low-glucose neuronal culture medium, containing 1:9 Neurobasal and Neurobasal-A medium (Thermo Fisher, #A2477501) supplemented with 1X B27 without vitamin A, 1X GlutaMAX Supplement (Thermo Fisher, #35050061), Pen/Strep, 20 ng ml^−1^ BDNF (R&D, #248-BD), 20 ng ml^−1^ GDNF (PeproTech, #450-10), 200 µM cyclic monophosphate sodium salt (cAMP; Sigma-Aldrich, #D0627) and 200 µM ascorbic acid (AA; Sigma-Aldrich, #4034-100). During replating, 10 μM Y-27632 was added to increase cell viability. Half of the medium was replaced every other day.

For experiments in mouse, B6.129_4 mouse epiblast stem cells (mEpiSCs) were cultured on Mouse Embryonic Fibroblast feeders (Thermo, #A34181) in mouse N2B27 (ref. ^80^) supplemented with 12.5 ng ml^−1^ FGF2, 20 ng ml^−1^ Activin A and 175 nM NVP-TNKS656. mEpiSCs were differentiated to neuroectodermal fate as follows: on day 0 mEpiSC colonies were lifted from feeders using 0.5 U µl^−1^ collagenase IV in HBSS++, dissociated to single cells using Accutase, then plated at 400,000 cells per cm^2^ on Matrigel-coated plates in mouse N2B27 supplemented with CEPT cocktail (50 nM chroman I, 5 µM Emricasan, 1:1,000 Poly amines, 0.7 µM Trans-ISRIB), 100 nM LDN193189, 10 µM SB431542, 200 nM LGK-974 and 2 µM cyclopamine. On days 1–3, cells were fed daily with the same media as day 0, excluding the CEPT cocktail. On days 4–6, cells were fed with mouse N2B27 supplemented with 2 µM cyclopamine only.

### Genome-wide CRISPR-Cas9 screens

The Brunello human CRISPR Knockout Pooled Library^26^ was used for this screen. Three hundred million PAX6::H2B-GFP iCas9 hESCs were infected with the lentiviral library at an MOI of 0.3 in a total of 31 × 100-mm plates for 20 hours. The infected cells were then selected by adding 0.4 μg ml^−1^ puromycin to the E8 medium for 4 days. After selection, one billion hESCs were dissociated with Accutase, pooled from all cultural plates, and cryopreserved in STEM-CELLBANKER freezing media (Amsbio #11924). To perform each replicate of the screen, two hundred million hESCs were thawed and plated at a density of 120,000–150,000 cells per cm^2^ in E8 medium with 10 μM Y-27632. To induce Cas9 expression, hESCs were treated with 1 μg ml^−1^ doxycycline for 48 hours. They were maintained in the E8 medium for another 24 hours before harvesting for differentiation. hESCs were differentiated as described in the ‘Neural differentiation’ subsection. After 72–84 hours of neural induction, cells were dissociated using Accutase and sorted using FACSAria according to GFP expression. PAX6-GFP^high^ population gate was established based on day 0 ESC, and an equal number of PAX6-GFP^low^ cells were sorted for comparison. One replicate was collected at 72 hours (3 days) and two replicates were collected at 84 hours (3.5 days). Day 0 ESC, PAX6-GFP^high^ and PAX6-GFP^low^ cells were pelleted and snap-frozen. Cell pellets were lysed, and genomic DNA was extracted (Qiagen) and quantified by Qubit (Thermo Scientific). A quantity of gDNA covering 1,000× representation of sgRNAs was PCR amplified to add Illumina adapters and multiplexing barcodes. Amplicons were quantified by Qubit and Bioanalyzer (Agilent) and sequenced on Illumina HiSeq 2500. Sequencing reads were aligned to the screened library and counts were obtained for each gRNA.

### Data analysis for pooled CRISPR screen

Sequencing reads were aligned to the screened library and the CRISPR screen was analysed using the edgeR pipeline as previously described^31^. *Z*-score was calculated for each gene as previously described^81^. gRNA enrichment was calculated by comparing PAX6-GFP^high^ to PAX6-GFP^low^ populations. To identify candidate hits, we removed essential genes and genes with only one gRNA recovered. Essential genes were defined as genes significantly (*P*-value < 0.05) depleted in both PAX6-GFP^high^ versus day 0 and PAX6-GFP^low^ versus day 0 comparisons (Supplementary Table 2). Genes with a *Z*-score>1 and *P*-value < 0.05 were considered hits and followed up with secondary validation experiments.

### DepMap analysis

We analysed the DepMap 22Q2 Public+Score, Chronos dataset that contains the genetic perturbation scores of 18,018 genes in 957 cancer cell lines^82^. We curated a list of top 27 screen hits and included top five correlates of each gene using Pearson correlation coefficients from DepMap gene essentiality scores above a minimum threshold (*r* > 0.25), resulting in a list of 96 genes. Correlation matrix heat map and network diagram were generated as previously described^83^. Gene cluster that includes the most candidate hits was shown.

### Secondary validation

Top scoring gRNAs of the top 27 candidate genes from the pooled screen were selected for the genetic approach in the secondary validation. gRNAs were cloned into the lentiGuide-Puro plasmid (Addgene #52963) as described by the Zhang lab^84^. PAX6::H2B-GFP iCas9 hESCs were infected with the gRNAs individually, selected using puromycin for 4 days, and Cas9 expression was induced by doxycycline for 2 days. Cells were differentiated as described in the ‘Neural differentiation’ subsection in array in 96-well plates and continuously imaged every 6 hours for 10 days by an imaging reader (Agilent BioTek Cytation 7). In order to image GFP signal, E6 medium was replaced by a customized DMEM/F12 (without riboflavin, pyridoxine hydrochloride, and phenol red) made by the MSKCC Media Preparation Core, supplemented 10 mg ml^−1^ transferrin (Sigma-Aldrich, #T0665), 5 mg ml^−1^ insulin (Sigma-Aldrich, #91077 C), 500 μM sodium selenite (Sigma-Aldrich, #S5261), 221 mM l-Ascorbic acid 2-phosphate sesquimagnesium salt hydrate (Sigma-Aldrich, #A8960), and 54.3 mg ml^−1^ sodium bicarbonate (Sigma-Aldrich, #S5761). For pharmacological approach, each small molecule inhibitor was tested across a logarithmic dose range, and the highest concentration that did not induce toxicity was selected for use in the differentiation assays. Small-molecule inhibitors were added to culture medium starting from day 0 and maintained throughout the neuroectoderm differentiation. A list of small molecule inhibitors, their molecular targets and concentrations used is reported in Supplementary Table 3.

### RNA-seq sample processing and analysis

Phase separation in cells lysed in TRIzol was induced with 200 µl chloroform. RNA was extracted from the aqueous phase using the MagMAX *mir*Vana Total RNA Isolation (Thermo Fisher, #A27828) on the KingFisher Flex Magnetic Particle Processor (Thermo Scientific, #5400630) according to the manufacturer’s protocol with 350 µl input. After RiboGreen quantification and quality control by Agilent BioAnalyzer, 500 ng of total RNA with RIN values of 9.3–10 underwent polyA selection and TruSeq library preparation according to instructions provided by Illumina (TruSeq Stranded mRNA LT Kit, #RS-122-2102), with eight cycles of PCR. Samples were barcoded and run on a NovaSeq 6000 in a PE100 run, using the NovaSeq 6000 S4 Reagent Kit (200 cycles) (Illumina). An average of 34 million paired reads was generated per sample. Ribosomal reads represented 0.17–0.52% of the total reads generated and the percentage of mRNA bases averaged 86%. The R package Rsubread^85^ was used to map reads to the human genome (GRCh38) using the align() function. Rsubread was also used to compute the expression count matrix from the mapped reads (BAM files) using the featureCounts() function. The R package DESeq2^86^ used to normalize the raw counts (median of ratios method) and perform differential gene expression analysis. Data are presented on a gene-by-gene basis as normalized counts or after carrying out a variance-stabilizing transformation followed by PCA analysis using the top 500 variable features.

### TCseq time course analysis

TCseq analysis was performed using the TCseq R package v1.28.0. RNA-seq normalized count table of NT day 0 to 10 data was used. Clustering was performed using c-means clustering by increasing cluster number until redundant clusters arose. To identify the effect of genotype in average time-variant signature, the averaged *Z* scores of selected timepoints were used as the aggregate gene expression signal as a function of time, then underwent nested, multivariate linear regression, contrasting the models with and without considering the effect of genetic KOs. The KO effects of *MEN1* and *SUZ12* were compared separately with the NT control. *P-*values were calculated from ANOVA of *F* tests.

### Gene set enrichment analysis

GSEA was performed using GSEA software v4.3.3. For pathway analysis in the pooled screen, a ranked list of 18,897 genes recovered from the screen, ranked by their *Z*-scores, was used as input for GSEAPreranked. C5.go.v2024.1.Hs.symbols gene set database was used. Top 100 positive pathways were visualized using ggplot2. For enrichment analysis in the NT versus KO comparisons, normalized count table was used as the expression dataset. Gene clusters identified from TCseq were used as gene sets database. Comparisons were done on *SUZ12* KO day 4 versus NT day 4 and *MEN1* KO day 4 versus NT day 4.

### ATAC-seq sample processing and analysis

Two clones were used for each KO line and two biological replicates were collected. Profiling of chromatin was performed by ATAC-Seq as previously described^46^. Briefly, 50,000–100,000 frozen hESC were thawed, washed in cold PBS and lysed. The transposition reaction containing TDE1 Tagment DNA Enzyme (Illumina, #20034198) was incubated at 37 °C for 30 minutes. The DNA was cleaned with the MinElute PCR Purification Kit (QIAGEN, #28004) and material was amplified for five cycles using NEBNext High-Fidelity 2X PCR Master Mix (New England Biolabs, #M0541L). After evaluation by real-time PCR, 1–3 additional PCR cycles were done. The final product was cleaned by aMPure XP beads (Beckman Coulter, #A63882) at a 1.0× ratio, and size selection was performed at a 1.5× ratio. Libraries were sequenced on a NovaSeq 6000 X in a PE100 run, using the NovaSeq 6000 S1 and S4 Reagent Kits (200 Cycles) (Illumina). An average of 52 million paired reads were generated per sample. Sequencing data were aligned to the hg38 reference genome using bowtie2 (v2.5.0)^87^. Ends of the aligned reads were shifted to remove Tn5 transposase artefacts as previously described^46,88^. Macs2 (v2.2.7.1)^89^ was used to remove duplicate reads and to call peaks, with the options extending the reads to both sides, and using a permissive *P*-value threshold (-p 1e-2–nomodel–shift −100–extsize 200–scale-to large–keep-dup all). If replicates were available, peaks were called at the combined level and the replicate level. They were then filtered using irreproducible discovery rate (IDR) (v2.0.4.2)^90^ using the replicates with the threshold of 0.01. If a sample was not prepared in replicates, peaks at the combined level were filtered where the RPKM quantification is above 1.85 in at least one sample. The signal intensity of the samples was first written in raw counts and normalized by library size factor defined by DESeq2’s median normalization. Differentially accessible peaks were assigned using DESeq2 (v1.39)^86^ using the *P*_adj_ threshold of 0.05.

### CUT&RUN sample processing and analysis

One clone was used for each KO line and two biological replicates were collected. CUT&RUN was performed from 100,000 cells per condition using the following antibodies at 1:100 dilution: rabbit anti-H3K4me3 (Abcam, #ab8580); rabbit anti-H3K27me3 (Cell Signaling Technologies, #9733); rabbit anti-Menin (Cell Signaling Technologies, #6891); rabbit anti-MLL1 (Cell Signaling Technologies, #14689); rabbit anti-SUZ12 (Cell Signaling Technologies, #3737); normal rabbit IgG (Cell Signaling Technologies, #2729). In brief, cells were permeabilized with digitonin and the different antibodies were incubated overnight at 4 °C on a rotator. Cells were washed and incubated with pA-MN. Beads Ca^2+^-induced digestion occurred on ice for 30 min and stopped by chelation. DNA was isolated using an extraction method with phenol and chloroform as previously described^47^. Immunoprecipitated DNA was quantified by PicoGreen and the size was evaluated by Agilent BioAnalyzer. When possible, fragments between 100 and 600 bp were size selected using aMPure XP beads (Beckman Coulter, #A63882) and Illumina sequencing libraries were prepared using the KAPA HTP Library Preparation Kit (Kapa Biosystems KK8234) according to the manufacturer’s instructions with 0.3–5 ng input DNA and 14 cycles of PCR. Barcoded libraries were run on the NovaSeq 6000 in a PE100 run, using the NovaSeq 6000 S4 Reagent Kit (200 Cycles) (Illumina). An average of 15 million paired reads were generated per sample. Sequencing data were aligned to the hg38 reference genome using bowtie2 (v2.5.1). Macs2 (v2.2.7.1) was executed to remove duplicate reads and to call peaks with the respective input control and using a permissive *P*-value threshold. Peaks were further filtered by IDR, with the threshold chosen as 0.01 for H3K4me3 and 0.05 for H3K27me3. The signal intensity of the samples was written during the peak calling (-B -p 1e-2–scale-to large–keep-dup all). It was then normalized by library size factor defined by DESeq2’s median normalization and converted to bigWig format. Deeptools^91^ was used for heatmaps and gene-profile plots. Tracks of CUT&RUN peaks were visualized using Integrative Genomics Viewer v2.16.1 (IGV, Broad Institute) or trackplot^92^ R script. ChIP-seq data in mEpiSCs were downloaded from the ChIP-Atlas database^93^ with the threshold chosen as 100 or 500.

### Definitive endoderm differentiation

Definitive endoderm differentiation was performed as previously described^94^. hESCs were dissociated into single cells using Accutase and plated at 125,000 cells per cm^2^ onto vitronectin-coated plates in E8 medium with 10 μM ROCK inhibitor Y-27632 (day −1). The next day (day 0), cells were washed with 1xDPBS once and culture medium was changed to Advanced RPMI (Thermo Fisher Scientific, #12633012) with penicillin streptomycin and 1X GlutaMAX, supplemented with 50 ng ml^−1^ Activin A (R&D systems, #338-AC-MTO) for 3 days (day 0–2) and 5 μM CHIR99021 (R&D systems, #4423/50) for the first day (day 0). Marker expression was assessed using flow cytometry and gating was established using hESCs as negative control.

### Cardiomyocyte differentiation

Cardiomyocyte differentiation was performed as previously described^55^ with some modifications. hESCs were dissociated into single cells using Accutase and plated at 80,000 cells per cm^2^ onto matrigel-coated plates in E8 medium with 10 μM ROCK inhibitor Y-27632 (day −2). Cells were maintained in E8 medium for another day (day −1) before changing to base medium containing RPMI 1640 medium (Fisher Scientific, #MT10041CV) and 1X B27 supplement minus insulin (Fisher Scientific, #A1895601) or Advanced RPMI (Thermo Fisher Scientific, #12633012) with Penicillin Streptomycin and 1X GlutaMAX, supplemented with 5 μM CHIR99021 (day 0). On day 1, CHIR99021 was removed, and culture medium was changed into base medium. From day 2 to 3, cells were treated with IWP-2 (Stemgent, #04-0034) in the base medium for 48 hours. From day 4 onwards, IWP-2 was removed, and base medium was changed every other day. Marker expression was assessed using flow cytometry and gating was established using hESCs as negative control. Contraction was assessed in 96-well plates using an imaging reader (Agilent BioTek Cytation 7). A 5-second video with 20 fps acquisition speed and 1,390 × 1,390 μm field-of-view was recorded for each well and contraction phenotype was observed by eye and counted manually. The position of imaged area was kept constant across all wells and at least six wells were assessed for each condition.

### Small-molecule treatments

The following small molecules targeting EZH2 and Menin–MLL interaction were used in the study: Tazemetostat (EPZ-6438) (Selleckchem, #S7128); VTP50469 (Selleckchem, #S8934). The following doses were used: for definitive endoderm differentiation and neurogenesis, 0.025% DMSO, 2.5 μM VTP50469, and 2.5 μM Tazemetostat; for cardiomyocyte differentiation and neuroectoderm differentiation of mEpiSCs, 0.0125% or 0.025% DMSO, 1.25 μM VTP50469, and 1.25 μM tazemetostat. For human neuroectoderm, definitive endoderm, cardiomyocyte and mouse neuroectoderm differentiation, small-molecule inhibitors were added to the culture medium starting from day 0 and maintained throughout the differentiation. For neurogenesis, small-molecule inhibitors were added to the culture media starting from day 20 and maintained throughout the differentiation.

### Protein extraction and western blot

Cells were collected and lysed in RIPA buffer (Sigma) with 1:100 Halt Protease and Phosphatase Inhibitor Cocktail (Thermo Fisher Scientific) and then sonicated for 3 × 30 sec at 4 °C. Protein lysates were centrifugated for 15 min at more than 15,000*g* at 4 °C and supernatant was collected and quantified by Precision Red Advanced Protein Assay (Cytoskeleton). Protein (5–10 μg) was boiled in NuPAGE LDS sample buffer (Invitrogen) at 95 °C for 5 min and separated using NuPAGE 4–12% Bis-Tris Protein Gel (Invitrogen) in NuPAGE MES SDS Running Buffer (Invitrogen). Proteins were electrophoretically transferred to nitrocellulose membranes (Thermo Fisher Scientific) with NuPAGE Transfer Buffer (Invitrogen). Blots were blocked for 60 min at room temperature in TBS-T + 5% nonfat milk (Cell Signaling Technologies) and incubated overnight in the same solution with the respective primary antibodies at 4 °C. The following primary antibodies were used at 1:500 dilution: mouse anti-Cas9 (Cell Signaling Technologies, #14697S); rabbit anti-Menin (EpiCypher, #13-2021); rabbit anti-SUZ12 (Cell Signaling Technologies, #3737S); HRP-conjugated rabbit anti-GAPDH (Cell Signaling Technologies, #3683S). The following secondary antibodies were incubated for 1 h at room temperature at 1:1,000 dilution: anti-mouse IgG HRP-linked (Cell Signaling Technologies, #7076); anti-rabbit IgG HRP-linked (Cell Signaling Technologies, #7074). Blots were revealed using SuperSignalTM West Femto Chemiluminescent Substrate (Thermo Fisher Scientific) at ChemiDoc XRS+ system (Bio-Rad). Chemiluminescence was imaged and analysed using Image lab software version 6.1.0 (Bio-Rad).

### RNA extraction and RT-qPCR

Cells were collected in Trizol and processed using Direct-zol RNA Miniprep kit (Zymo Research, # R2050). A total of 1 μg of RNA was used to generate cDNA using iScript (Bio-Rad). Real-time PCR was performed using SsoFAST EvaGreen Mix (Bio-Rad) in a Bio-Rad CFX96 Thermal Cycler. The manufacturers protocol was used for all steps. Samples in which the housekeeping gene had a cycle threshold (Ct) value above 30 were repeated. Primer sequences used in the study are listed in the Supplementary Table 5.

### Immunofluorescence staining

Cultured cells were fixed with 4% PFA in PBS for 20 min at room temperature, washed three times with PBS, permeabilized for 30 min in 0.5% Triton X-100 in PBS and then blocked in a solution containing 5% Normal donkey serum, 2% BSA and 0.25% Triton X-100 for 1 hour at room temperature. Primary antibodies were incubated overnight at 4 °C in the same blocking solution. Secondary antibodies conjugated to either Alexa 488, Alexa 555 or Alexa 647 (Thermo) were incubated for 45 min at 1:400 dilution in blocking solution. Cell nuclei were stained with 5 μM 4′-6-diamidino-2-phenylindole (DAPI) in PBS. The following primary antibodies and dilutions were used: mouse anti-HuC/HuD 1:500 (Thermo Scientific, #A-21271); chicken anti-MAP2 1:2,000 (Abcam, # ab5392); rat anti-SOX2 1:500 (Thermo Scientific, #14-9811-82). To quantify the percentage of positive cells, images were converted to 8-bit and a threshold was set for DAPI to exclude bright cells. ‘Analyse Particles’ was used to quantify the total number of cells. A threshold was set for each marker, and cells with over 50% of DAPI area that met the threshold requirement were counted as positive. Thresholds were kept constant across all wells per replicate. 6–10 images of were taken from 3 individual wells for each condition and averaged.

### Flow cytometry

Cells were dissociated to single cells using Accutase. For live cell staining, cells were incubated with conjugated antibodies in 3% fetal bovine serum (FBS) in PBS for 45 min at 4 °C. Cell nuclei were stained with DAPI in PBS, and live cells were gated based on negative DAPI signal. Fixed cell staining was performed using BD Cytofix/Cytoperm kit (BD Biosciences, #554714) according to manufacturer’s instructions. The following antibodies were used at 1:100 dilution: Alexa 488 mouse anti-PAX6 (BD Biosciences, #561664); PE-CF594 mouse anti-PLZF (ZBTB16) (BD Biosciences, #565738); Alexa 647 mouse anti-CXCR4 (Biotechne, # FAB172R-100UG); PE rabbit anti-GATA6 (Cell Signaling Technologies, #26452S); APC goat anti-SOX17 (Biotechne, #IC1924A); PE mouse anti-SIRP alpha (R&D systems, #FAB4546P); APC rat anti-CD324 (E-Cadherin) (Biolegend, #147311); PE Mouse Anti-Cardiac Troponin T (BD Biosciences, #564767). Results were analysed using FlowJo. Mean fluorescence intensity (MFI) of markers assessed by flow cytometry is reported in the Supplementary Table 6.

### Statistics and reproducibility

No statistical method was used to predetermine sample sizes, but our sample sizes are similar to those reported in the previous publications^95,96^. Data collection and analysis were not performed blind to the conditions of the experiments. For secondary validation experiments, samples were de-identified respect to the molecular or genetic target and a number code was assigned for each condition. No randomization was involved in the differentiation assays. No data were excluded from the analyses unless the differentiation experiment itself failed. Statistical analysis was carried out using GraphPad Prism 10. The details of statistical analyses and significance values are specified in the figures and figure legends. Data were reported as the mean ± s.d. or s.e.m, or independent replicates shown as individual data points. Data distribution was assumed to be normal, but this was not formally tested. For statistical analyses conducted using one-way ANOVA test with post-hoc Dunnett’s multiple comparisons test, results from one-way ANOVA test with post-hoc Tukey’s multiple comparisons test are provided in the Supplementary Table 7.

### Reporting summary

Further information on research design is available in the Nature Portfolio Reporting Summary linked to this article.

## Online content

Any methods, additional references, Nature Portfolio reporting summaries, source data, extended data, supplementary information, acknowledgements, peer review information; details of author contributions and competing interests; and statements of data and code availability are available at 10.1038/s41556-025-01751-5.

## Supplementary information

Reporting Summary

Supplementary Tables Supplementary Tables 1–7.

## Source data

Source Data Figs. 1–5 and Extended Data Figs. 1–9 Statistical source data.

Source Data Extended Data Figs. 1 and 5 Unprocessed blots.

## Figures and Tables

**Extended Data Fig. 10:**
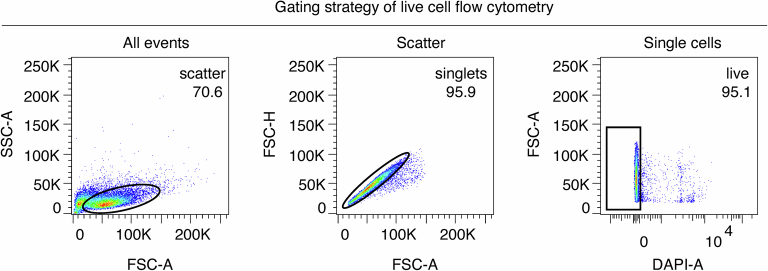
Gating strategy of live cell flow cytometry. Single cells are sorted based on FSC-A/SSC-A and FSC-A/FSC-H signal, then live cells are sorted based on DAPI signal.

## Data Availability

Sequencing data supporting the findings of this study have been deposited in the Gene Expression Omnibus (GEO) under accession code GSE279036. Previously published PAX6 binding data that were re-analysed here are available under accession code GSE216477. Source data are provided with this paper. Unprocessed flow cytometry samples and immunostaining images supporting the main findings of this study are available at 10.5281/zenodo.15795406 (ref. ^97^). All other data supporting the findings of this study are available from the corresponding authors upon reasonable request. Source data are provided with this paper.
