# Supplementary material for: Genome-wide CRISPR screen identifies Menin and SUZ12 as regulators of human developmental timing
Source: Nat Cell Biol. 2025 Sep 2;27(9):1411–21. doi: 10.1038/s41556-025-01751-5 (PMC12431854; doi:10.1038/s41556-025-01751-5)
Supplement: Supplementary file 1 — Reporting Summary [file 41556_2025_1751_MOESM1_ESM.pdf]

nature portfolio

Corresponding author(s): Lorenz Studer

Last updated by author(s): Jul 2, 2025

Reporting Summary

Nature Portfolio wishes to improve the reproducibility of the work that we publish. This form provides structure for consistency and transparency in reporting. For further information on Nature Portfolio policies, see our Editorial Policies and the Editorial Policy Checklist.

Statistics

For all statistical analyses, confirm that the following items are present in the figure legend, table legend, main text, or Methods section.

n/a	Confirmed	
☐	☒	The exact sample size (*n*) for each experimental group/condition, given as a discrete number and unit of measurement
☐	☒	A statement on whether measurements were taken from distinct samples or whether the same sample was measured repeatedly
☐	☒	The statistical test(s) used AND whether they are one- or two-sided<br>*Only common tests should be described solely by name; describe more complex techniques in the Methods section.*
☒	☐	A description of all covariates tested
☐	☒	A description of any assumptions or corrections, such as tests of normality and adjustment for multiple comparisons
☐	☒	A full description of the statistical parameters including central tendency (e.g. means) or other basic estimates (e.g. regression coefficient) AND variation (e.g. standard deviation) or associated estimates of uncertainty (e.g. confidence intervals)
☐	☒	For null hypothesis testing, the test statistic (e.g. *F*, *t*, *r*) with confidence intervals, effect sizes, degrees of freedom and *P* value noted<br>*Give P values as exact values whenever suitable.*
☒	☐	For Bayesian analysis, information on the choice of priors and Markov chain Monte Carlo settings
☒	☐	For hierarchical and complex designs, identification of the appropriate level for tests and full reporting of outcomes
☐	☒	Estimates of effect sizes (e.g. Cohen's *d*, Pearson's *r*), indicating how they were calculated

Our web collection on statistics for biologists contains articles on many of the points above.

Software and code

Policy information about availability of computer code

Data collection	Image Lab (Bio-Rad) version 6.1.0 was used for Western Blot image acquisition; ZEN Blue 3.1 was used for immunofluorescence imaging; BioTek Gen5 was used for live cell imaging; BD FACSDiva was used for flow cytometry; BioRad CFX Maestro was used for RT qPCR.
Data analysis	FlowJo v10 was used for flow cytometry analysis; Integrative Genomics Viewer v2.16.1 was used to visualize for viewing epigenomic tracks; GSEA software v4.3.3 was used for Gene Set enrichment Analysis (GSEA); edgeR v4.2.1 was used for genetic screen analysis; FastQC v0.11.9, Rsubread v1.30.9, IDR v2.0.4.2, DESeq2 v1.39, Macs2 v2.2.7.1, bowtie2 v2.5.0, and Deeptools v3.5.5 were used for bioinfomatic analyses of RNAseq, ATAC-seq and CUT&RUN data; TCseq v1.28.0 was used for time-course analysis of RNA-seq; R version 4.4.0 and GraphPad Prism 10 were used for statistical computing and graphing.

For manuscripts utilizing custom algorithms or software that are central to the research but not yet described in published literature, software must be made available to editors and reviewers. We strongly encourage code deposition in a community repository (e.g. GitHub). See the Nature Portfolio guidelines for submitting code & software for further information.

Data

Policy information about availability of data

All manuscripts must include a data availability statement. This statement should provide the following information, where applicable:

- Accession codes, unique identifiers, or web links for publicly available datasets
- A description of any restrictions on data availability
- For clinical datasets or third party data, please ensure that the statement adheres to our policy

Sequencing data supporting the findings of this study have been deposited in the Gene Expression Omnibus (GEO) under accession code GSE279036. Previously published PAX6 binding data that were re-analyzed here are available under accession code GSE216477. Source data are provided with this paper. All other data supporting the findings of this study are available from the corresponding authors upon reasonable request.

Research involving human participants, their data, or biological material

Policy information about studies with human participants or human data. See also policy information about sex, gender (identity/presentation), and sexual orientation and race, ethnicity and racism.

Reporting on sex and gender	No human participants were involved in this study.
Reporting on race, ethnicity, or other socially relevant groupings	*Please specify the socially constructed or socially relevant categorization variable(s) used in your manuscript and explain why they were used. Please note that such variables should not be used as proxies for other socially constructed/relevant variables (for example, race or ethnicity should not be used as a proxy for socioeconomic status).* *Provide clear definitions of the relevant terms used, how they were provided (by the participants/respondents, the researchers, or third parties), and the method(s) used to classify people into the different categories (e.g. self-report, census or administrative data, social media data, etc.)* *Please provide details about how you controlled for confounding variables in your analyses.*
Population characteristics	*Describe the covariate-relevant population characteristics of the human research participants (e.g. age, genotypic information, past and current diagnosis and treatment categories). If you filled out the behavioural & social sciences study design questions and have nothing to add here, write "See above."*
Recruitment	*Describe how participants were recruited. Outline any potential self-selection bias or other biases that may be present and how these are likely to impact results.*
Ethics oversight	*Identify the organization(s) that approved the study protocol.*

Note that full information on the approval of the study protocol must also be provided in the manuscript.

Field-specific reporting

Please select the one below that is the best fit for your research. If you are not sure, read the appropriate sections before making your selection.

☒ Life sciences ☐ Behavioural & social sciences ☐ Ecological, evolutionary & environmental sciences

For a reference copy of the document with all sections, see nature.com/documents/nr-reporting-summary-flat.pdf

Life sciences study design

All studies must disclose on these points even when the disclosure is negative.

Sample size	No statistical methods were used to predetermine sample sizes. Sample sizes were estimated based on previous experience and previous publications in the field. (Qi et al., Nat Biotechnol 2017; Tchieu et al., Nat Biotechnol 2019).
Data exclusions	No samples were excluded unless the differentiation itself failed.
Replication	CRISPR screens and RNA-seq experiments were performed with 3 biological replicates. Secondary validation genetic experiments were performed with 2-4 replicates, and pharmacological experiments were performed with 3 replicates. ATAC-seq and CUT&RUN experiments were performed with 2 biological replicates. The number of replicates for lineage differentiation assays is specified in the figure legend. Differentiation experiments where cells detached from plates were not included as replicates.
Randomization	For secondary validation experiments, samples were de-identified respect to the molecular or genetic target and a number code was assigned for each condition. No randomization was involved in the differentiation assays. The investigators designed, performed and analyzed these experiments, so randomization was not applicable to these experiments.
Blinding	No blinding was involved. The investigators designed, performed and analyzed the experiments, so blinding was not applicable to these experiments. Experimental results were generated by equipment- or software-based quantitative measurements rather than subjective rating of data that could be affected by no blinding.

Reporting for specific materials, systems and methods

We require information from authors about some types of materials, experimental systems and methods used in many studies. Here, indicate whether each material, system or method listed is relevant to your study. If you are not sure if a list item applies to your research, read the appropriate section before selecting a response.

Materials & experimental systems

n/a	Involved in the study
☐	☒ Antibodies
☐	☒ Eukaryotic cell lines
☒	☐ Palaeontology and archaeology
☒	☐ Animals and other organisms
☒	☐ Clinical data
☒	☐ Dual use research of concern
☒	☐ Plants

Methods

n/a	Involved in the study
☐	☒ ChIP-seq
☐	☒ Flow cytometry
☒	☐ MRI-based neuroimaging

Antibodies

Antibodies used

Primary antibodies:
mouse anti-Cas9 (Cell Signalling Tech, #14697S) at 1:1000 dilution for Western Blot
rabbit anti-Menin (EpiCypher, #13-2021) at 1:400 dilution for immunofluorescence and at 1:1000 for Western Blot
rabbit anti-SUZ12 (Cell Signalling Tech, #3737S) at 1:400 dilution for immunofluorescence, at 1:100 dilution for CUT&RUN, and at 1:1000 for Western Blot
mouse anti-PAX6 (BD Biosciences, #561462) at 1:400 dilution for immunofluorescence
mouse anti-HuC/HuD (Thermo Scientific, #A-21271) at 1:400 dilution for immunofluorescence
rat anti-SOX2 (Thermo Scientific, #14-9811-82) at 1:400 dilution for immunofluorescence
chicken anti-MAP2 (Abcam, # ab5392) at 1:1000 dilution for immunofluorescence
rabbit anti-H3K4me3 (Abcam, #ab8580) at 1:100 dilution for CUT&RUN
rabbit anti-H3K27me3 (Cell Signaling Technologies, #9733) at 1:100 dilution for CUT&RUN and at 1:400 for immunofluorescence
rabbit anti-Menin (Cell Signaling Technologies, #6891) at 1:100 dilution for CUT&RUN
rabbit anti-MLL1 (Cell Signaling Technologies, #14689) at 1:100 dilution for CUT&RUN
normal rabbit IgG (Cell Signaling Technologies, #2729) at 1:100 dilution for CUT&RUN

Secondary antibodies:
anti-mouse IgG HRP-linked (Cell Signaling Tech, #7076) at 1:5000 dilution for Western Blot
anti-rabbit IgG HRP-linked (Cell Signaling Tech, #7074) at 1:5000 dilution for Western Blot
donkey anti-mouse Alexa Fluor 555 (ThermoFisher Scientific, #A31570) at 1:500 dilution for immunofluorescence
goat anti-chicken Alexa Fluor 647 (ThermoFisher Scientific, #A21449) at 1:500 dilution for immunofluorescence

Conjugated antibodies:
HRP-conjugated rabbit anti-GAPDH (Cell Signalling Tech, #3683S) at 1:1000 dilution for Western Blot
Alexa488 mouse anti-PAX6 (BD Biosciences, #561664) at 1:50 dilution for flow cytometry
Alexa647 mouse anti-CXCR4 (Biotechne, # FAB172R-100UG) at 1:50 dilution for flow cytometry
PE rabbit anti-GATA6 (Cell Signalling Tech, #26452S) at 1:50 dilution for flow cytometry
PE mouse anti-SIRP alpha (R&D systems, #FAB4546P) at 1:50 dilution for flow cytometry
PE-CF594 mouse anti-PLZF (ZBTB16) (BD Biosciences, #565738) at 1:50 dilution for flow cytometry
APC goat anti-SOX17 (Biotechne, #IC1924A) at 1:50 dilution for flow cytometry
APC rat anti-CD324 (E-Cadherin) (Biolegend, #147311) at 1:50 dilution for flow cytometry
PE Mouse Anti-Cardiac Troponin T (BD Biosciences, #564767) at 1:50 dilution for flow cytometry

Validation

All antibodies were commercially validated as below:

mouse anti-Cas9 (Cell Signalling Tech, #14697S)
https://www.cellsignal.com/products/primary-antibodies/cas9-s-pyogenes-7a9-3a3-mouse-mab/14697?srsltid=AfmBOopa__zC01emzpl95rItlpG4PpGa1329wGqOJB_CpIB8EPhrtc09

rabbit anti-Menin (EpiCypher, #13-2021)
Validation was performed by manufacturer using 500k K562 cells with 0.5 µg of either Menin or IgG negative control.
https://www.epicypher.com/products/antibodies/cutana-cut-run-antibodies/cut-run-antibodies-chromatin-associated-proteins/menin-cutana-cut-run-antibody

rabbit anti-SUZ12 (Cell Signalling Tech, #3737S)
https://www.cellsignal.com/products/primary-antibodies/suz12-d39f6-xp-rabbit-mab/3737?srsltid=AfmBOorypUPT1g328HovPi2ABgeXULnynFvRA3heIe7_5bHWF8uzDX-v

mouse anti-HuC/HuD 1:500 (Thermo Scientific, #A-21271)
Validation was performed by manufacturer in neurons differentiated from H9 ESC derived NSCs.

https://www.thermofisher.com/antibody/product/HuC-HuD-Antibody-clone-16A11-Monoclonal/A-21271

chicken anti-MAP2 1:2000 (Abcam, # ab5392)
Validation was performed by manufacturer for ICC and WB in mouse and rat brain tissue.
https://www.abcam.com/en-us/products/primary-antibodies/map2-antibody-ab5392

rabbit anti-H3K4me3 (Abcam, #ab8580)
Validated by manufacturer as ChIP Grade, suitable for ChIP, WB, IHC-P, ICC/IF, and reactivity validated in human.
https://www.abcam.com/en-us/products/primary-antibodies/histone-h3-tri-methyl-k4-antibody-chip-grade-ab8580

rabbit anti-H3K27me3 (Cell Signaling Technologies, #9733)
Validation was performed by manufacturer for WB, IHC, IF, Chip, C&R and reactivity validated in human and mouse.
https://www.cellsignal.com/products/primary-antibodies/tri-methyl-histone-h3-lys27-c36b11-rabbit-mab/9733?srsltid=AfmBOooXVa5b-14bB1qXHlBAycAThZO7YIZOhS4x68uDbvupzJOV7KGH

rabbit anti-Menin (Cell Signaling Technologies, #6891)
Validation was performed by manufacturer for WB and IF and reactivity validated in human and mouse.
https://www.cellsignal.com/products/primary-antibodies/menin-d45b1-xp-rabbit-mab/6891?srsltid=AfmBOooSGBaINUc50VXMAfzFNEJsAQJZn0lmav3JDDad67MxbRI6soa4

rabbit anti-MLL1 (Cell Signaling Technologies, #14689)
Validation was performed by manufacturer for WB, IP and C&R and reactivity validated in human and mouse.
https://www.cellsignal.com/products/primary-antibodies/mll1-d2m7u-rabbit-mab-amino-terminal-antigen/14689?srsltid=AfmBOoqYbu2D-3s2zclZd-sxrrk4MDpiGreATKzCDgm8_kkBZmcLPHb7

rabbit anti-SUZ12 (Cell Signaling Technologies, #3737)
Validation was performed by manufacturer for WB, IP, IF, ChIP, and C&R and reactivity validated in human and mouse.
https://www.cellsignal.com/products/primary-antibodies/suz12-d39f6-xp-rabbit-mab/3737?srsltid=AfmBOooS96dVD6Eusd78hh-0-Hai-iWUqnIkyHoANIYBcVqWj7Zt72Gm

rat anti-SOX2 (Thermo Scientific, #14-9811-82)
https://www.thermofisher.com/antibody/product/SOX2-Antibody-clone-Btjce-Monoclonal/14-9811-82

normal rabbit IgG (Cell Signaling Technologies, #2729)
Validation was performed by manufacturer for IP and ChIP.
https://www.cellsignal.com/products/primary-antibodies/normal-rabbit-igg/2729?srsltid=AfmBOor6PmyKMXJODabuVL5bM2KJREIMEwaJzzrsHybrqKd_ETy8WyJy

anti-mouse IgG HRP-linked (Cell Signaling Tech, #7076)
https://www.cellsignal.com/products/secondary-antibodies/anti-mouse-igg-hrp-linked-antibody/7076?srsltid=AfmBOorNWMYlOSaz8W12p-naYoTUCokYq5dZ5Grm8Z012q9mQeDafcLP

anti-rabbit IgG HRP-linked (Cell Signaling Tech, #7074)
https://www.cellsignal.com/products/secondary-antibodies/anti-rabbit-igg-hrp-linked-antibody/7074?srsltid=AfmBOopKoqlALhAyyUcuFbx6viZKDl0wvUhfZvpY3n_4TqJs2Qc9bzO9

donkey anti-mouse Alexa Fluor 555 (ThermoFisher Scientific, #A31570)
https://www.thermofisher.com/antibody/product/Donkey-anti-Mouse-IgG-H-L-Highly-Cross-Adsorbed-Secondary-Antibody-Polyclonal/A-31570

goat anti-chicken Alexa Fluor 647 (ThermoFisher Scientific, #A21449)
https://www.thermofisher.com/antibody/product/Goat-anti-Chicken-IgY-H-L-Secondary-Antibody-Polyclonal/A-21449

HRP-conjugated rabbit anti-GAPDH (Cell Signalling Tech, #3683S)
Validation was performed by manufacturer for WB and reactivity validated in human and mouse.
https://www.cellsignal.com/products/antibody-conjugates/gapdh-14c10-rabbit-mab-hrp-conjugate/3683?srsltid=AfmBOooC7yW7udNe9AkqR1asdFvxsTEhar5IfshVH1yxoac1YMAeW2SO

Alexa488 mouse anti-Pax6 (BD Biosciences, #561664)
Validation was performed by manufacturer for intracellular staining (flow cytometry) and reactivity validated in human.
https://www.bdbiosciences.com/en-us/products/reagents/flow-cytometry-reagents/research-reagents/single-color-antibodies-ruo/alexa-fluor-488-mouse-anti-human-pax-6.561664

Alexa647 mouse anti-CXCR4 (Biotechne, # FAB172R-100UG)
Validation was performed by manufacturer for flow cytometry and reactivity validated in human.
https://www.bio-techne.com/p/antibodies/human-cxcr4-alexa-fluor-647-conjugated-antibody-44716_fab172r

PE rabbit anti-GATA6 (Cell Signalling Tech, #26452S)
Validation was performed by manufacturer for intracellular staining (flow cytometry) and reactivity validated in human.
https://www.cellsignal.com/products/antibody-conjugates/gata-6-d61e4-xp-rabbit-mab-pe-conjugate/26452?srsltid=AfmBOoqTxaTYRk01tKQjOn1MTQvh0z4cP_t9mxsqOmGn_1PX8Xybnub_

PE mouse anti-SIRP alpha (R&D systems, #FAB4546P)
Validation was performed by manufacturer for flow cytometry, WB and ELISAs and reactivity validated in human.
https://www.rndsystems.com/products/human-sirpalpha-cd172a-pe-conjugated-antibody-602411_fab4546p

PE-CF594 mouse anti-PLZF (ZBTB16) (BD Biosciences, #565738)
https://www.bdbiosciences.com/en-eu/products/reagents/flow-cytometry-reagents/research-reagents/single-color-antibodies-ruo/pe-cf594-mouse-anti-plzf.565738?tab=product_details

APC goat anti-SOX17 (Biotechne, #IC1924A)
https://www.rndsystems.com/products/human-sox17-apc-conjugated-antibody_ic1924a

APC rat anti-CD324 (E-Cadherin) (Biolegend, #147311)
https://www.biolegend.com/de-at/products/apc-anti-mouse-human-cd324-e-cadherin-antibody-16412

PE Mouse Anti-Cardiac Troponin T (BD Biosciences, #564767)
https://www.bdbiosciences.com/en-us/products/reagents/flow-cytometry-reagents/research-reagents/single-color-antibodies-ruo/pe-mouse-anti-cardiac-troponin-t.564767?tab=product_details

Eukaryotic cell lines

Policy information about cell lines and Sex and Gender in Research

Cell line source(s)

H9 (WA-09): WiCell Stemcell Bank; PAX6::H2B-GFP hPSC line was generated in the Studer lab by targeting of H9 hPSC (Tchieu J et al, Cell Stem Cell 2017); PAX6::H2B-GFP iCas9 hPSC line was generated in the Studer lab by targeting of PAX6::H2B-GFP hPSC line; PAX6 KO H1 hPSC, inducible CRISPRa H9 hPSC, inducible CRISPRi H9 hPSC: Memorial Sloan Kettering Cancer Center/ Stem Cell Research Core; mEpiSC B6.129_ 4: Memorial Sloan Kettering Cancer Center/ Vierbuchen lab (Medina-Cano et al., Dev 2022).

Authentication

hPSC lines were authenticated by the standard short tandem repeat (STR) profiling by MSK Integrated Genomics Operation core facility. PAX6::H2B-GFP iCas9 hPSC line was validated by genomic PCR and Cas9 mRNA and protein expression by qRT-PCR and Western Blot respectively and screened for Karyotype banding.

Mycoplasma contamination

All cell lines were regularly tested negative for mycoplasma contamination.

Commonly misidentified lines
(See ICLAC register)

No commonly misidentified lines were used in this study.

Plants

Seed stocks

No plants were used in this study.

Novel plant genotypes

Describe the methods by which all novel plant genotypes were produced. This includes those generated by transgenic approaches, gene editing, chemical/radiation-based mutagenesis and hybridization. For transgenic lines, describe the transformation method, the number of independent lines analyzed and the generation upon which experiments were performed. For gene-edited lines, describe the editor used, the endogenous sequence targeted for editing, the targeting guide RNA sequence (if applicable) and how the editor was applied.

Authentication

Describe any authentication procedures for each seed stock used or novel genotype generated. Describe any experiments used to assess the effect of a mutation and, where applicable, how potential secondary effects (e.g. second site T-DNA insertions, mosiacism, off-target gene editing) were examined.

ChIP-seq

Data deposition

☒ Confirm that both raw and final processed data have been deposited in a public database such as GEO.

☒ Confirm that you have deposited or provided access to graph files (e.g. BED files) for the called peaks.

Data access links
May remain private before publication.

https://www.ncbi.nlm.nih.gov/geo/query/acc.cgi?acc=GSE279036

Files in database submission

GSM8559893 Menin_H9_d0_CUT&RUN_rep1
GSM8559894 MLL1_H9_d0_CUT&RUN_rep1
GSM8559895 SUZ12_H9_d0_CUT&RUN_rep1
GSM8559896 Menin_H9_d0_CUT&RUN_rep2
GSM8559897 MLL1_H9_d0_CUT&RUN_rep2
GSM8559898 SUZ12_H9_d0_CUT&RUN_rep2
GSM8559899 IgG_H9_d0_CUT&RUN_rep1
GSM8559900 H3K4me3_H9_d0_CUT&RUN_rep1
GSM8559901 H3K27me3_H9_d0_CUT&RUN_rep1
GSM8559902 H3K4me3_MEN1_d0_CUT&RUN_rep1
GSM8559903 H3K27me3_MEN1_d0_CUT&RUN_rep1
GSM8559904 H3K4me3_SUZ12_d0_CUT&RUN_rep1
GSM8559905 H3K27me3_SUZ12_d0_CUT&RUN_rep1
GSM8559906 IgG_H9_d4_CUT&RUN_rep1

GSM8559907 H3K4me3_H9_d4_CUT&RUN_rep1
GSM8559908 H3K27me3_H9_d4_CUT&RUN_rep1
GSM8559909 IgG_H9_d8_CUT&RUN_rep1
GSM8559910 H3K4me3_H9_d8_CUT&RUN_rep1
GSM8559911 H3K27me3_H9_d8_CUT&RUN_rep1
GSM8559912 IgG_H9_d0_CUT&RUN_rep2
GSM8559913 H3K4me3_H9_d0_CUT&RUN_rep2
GSM8559914 H3K27me3_H9_d0_CUT&RUN_rep2
GSM8559915 H3K4me3_MEN1_d0_CUT&RUN_rep2
GSM8559916 H3K27me3_MEN1_d0_CUT&RUN_rep2
GSM8559917 H3K4me3_SUZ12_d0_CUT&RUN_rep2
GSM8559918 H3K27me3_SUZ12_d0_CUT&RUN_rep2
GSM8559919 IgG_H9_d4_CUT&RUN_rep2
GSM8559920 H3K4me3_H9_d4_CUT&RUN_rep2
GSM8559921 H3K27me3_H9_d4_CUT&RUN_rep2
GSM8559922 IgG_H9_d8_CUT&RUN_rep2
GSM8559923 H3K4me3_H9_d8_CUT&RUN_rep2
GSM8559924 H3K27me3_H9_d8_CUT&RUN_rep2

Genome browser session (e.g. UCSC): Integrative Genomics Viewer v2.16.1 hg38

Methodology

Replicates: Two biological replicate experiments for each condition were performed.

Sequencing depth: An average of 15 million paired reads were generated per sample.

Antibodies: rabbit anti-H3K4me3 (Abcam, #ab8580); rabbit anti-H3K27me3 (Cell Signaling Technologies, #9733); rabbit anti-Menin (Cell Signaling Technologies, #6891); rabbit anti-MLL1 (Cell Signaling Technologies, #14689); rabbit anti-SUZ12 (Cell Signaling Technologies, #3737); normal rabbit IgG (Cell Signaling Technologies, #2729)

Peak calling parameters: Sequencing data were aligned to the hg38 reference genome using bowtie2 (v. 2.5.1). Macs2 (v. 2.2.7.1) was executed to remove duplicate reads and to call peaks with the respective input control and using a permissive P value threshold.

Data quality: Peaks were further filtered by IDR, with the threshold chosen as 0.01 for H3K4me3 and 0.05 for H3K27me3.

Software: Bowtie2 (v2.5.1), Macs2 (v2.2.7.1), IDR (v2.0.4.2), DESeq2 (v1.39) and Deeptools (v3.5.5) were used for the analysis of CUT&RUN data.

Flow Cytometry

Plots

Confirm that:

☒ The axis labels state the marker and fluorochrome used (e.g. CD4-FITC).

☒ The axis scales are clearly visible. Include numbers along axes only for bottom left plot of group (a 'group' is an analysis of identical markers).

☒ All plots are contour plots with outliers or pseudocolor plots.

☒ A numerical value for number of cells or percentage (with statistics) is provided.

Methodology

Sample preparation: Cells were dissociated to single cells using Accutase. For live cell staining, cells were incubated with conjugated antibodies in 3% fetal bovine serum (FBS) in PBS for 45 min at 4°C. Cell nuclei were stained with DAPI in PBS, and live cells were gated based on negative DAPI signal. Fixed cell staining was performed using BD Cytofix/Cytoperm kit (BD Biosciences, #554714) according to manufacturer's instructions.

Instrument: BD LSRFortessa

Software: FlowJo v10

Cell population abundance: Typically 10000 live cells were collected for flow cytometry analysis.

Gating strategy: Single cells were identified using sequential FSC-A/SSC-A, FSC-A/FSC-H, and SSC-A/SSC-H gating. Live cells were identified using DAPI staining. For gating controls in the differentiation assays, we used true negative cells, typically ESCs or day 0 cells collected prior to the differentiation.

☒ Tick this box to confirm that a figure exemplifying the gating strategy is provided in the Supplementary Information.
