# Supplementary material for: Genome-wide CRISPR screen identifies Menin and SUZ12 as regulators of human developmental timing
Source: Nat Cell Biol. 2025 Sep 2;27(9):1411–21. doi: 10.1038/s41556-025-01751-5 (PMC12431854; doi:10.1038/s41556-025-01751-5)

Unprocessed Western Blots

Ext. data fig. 1d

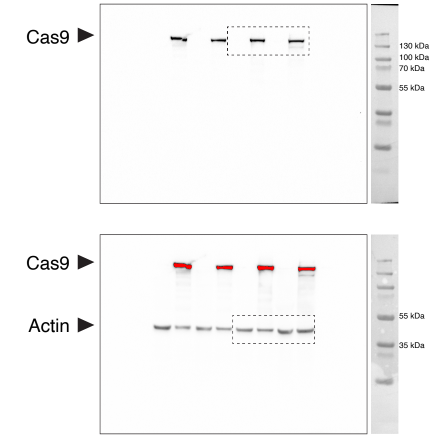

Ext. data fig. 5a

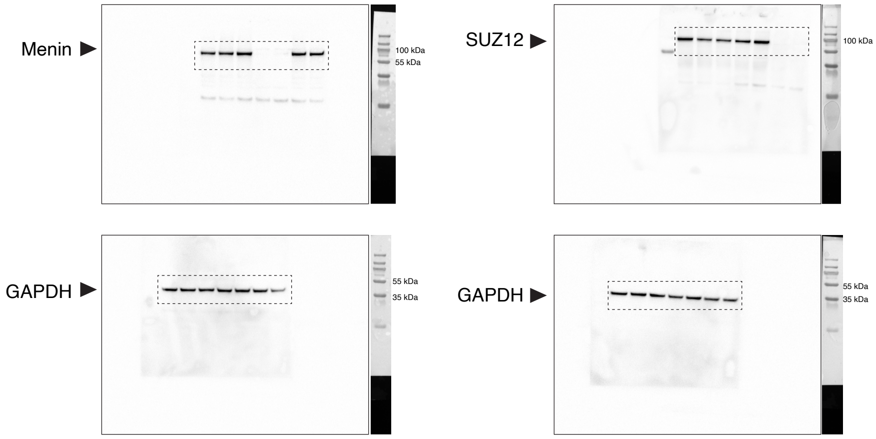

Ext. data fig. 7b

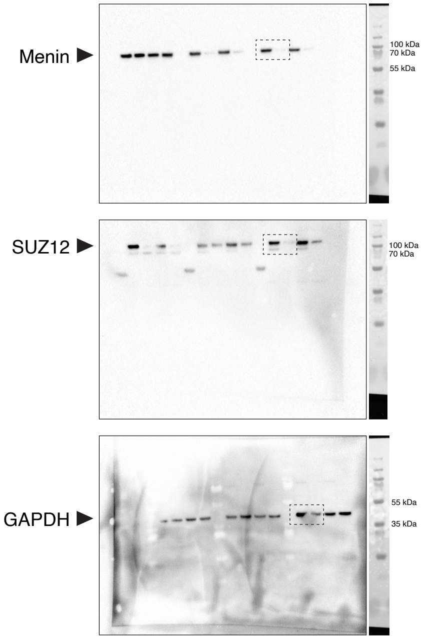

Ext. data fig. 9b

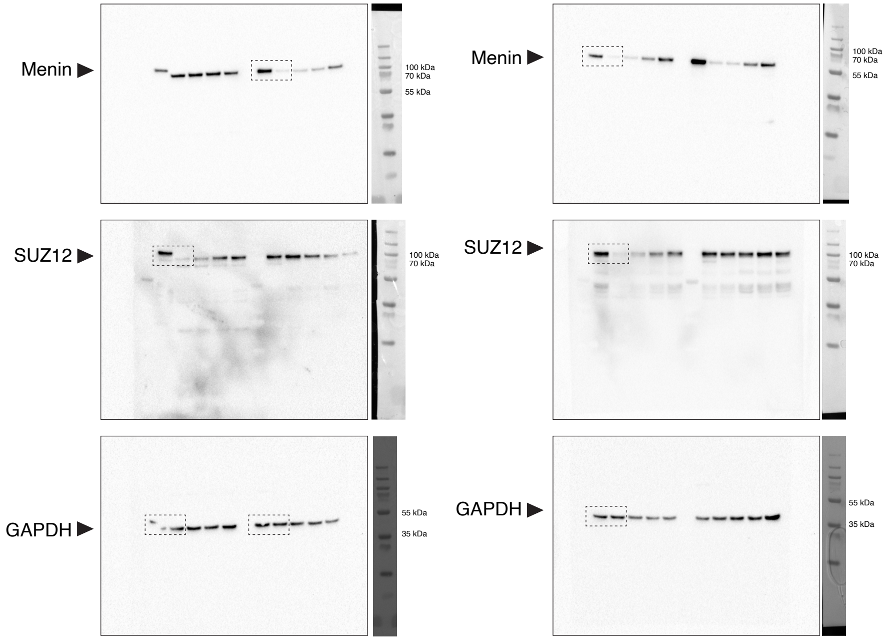

Supplement: Supplementary file 4 — Unprocessed blots. [file 41556_2025_1751_MOESM4_ESM.pdf]
